# Electronic Questionnaires Design and Implementation

**DOI:** 10.2174/1874434601711010157

**Published:** 2017-10-31

**Authors:** Clara Minto, Giulia Beltrame Vriz, Matteo Martinato, Dario Gregori

**Affiliations:** 1 Unit of Biostatistics, Epidemiology and Public Health, Department of Cardiac, Thoracic and Vascular Sciences, University of Padova, Padova, Italy; 2 Department of Obstetrics, Santa Chiara Hospital, Trento, Italy; 3 University Hospital, Gastroenterology, Padova, Padova, Italy

**Keywords:** Electronic questionnaires, Patients reported outcomes, App, Web survey design, Bias reduction techniques, Electronic tools

## Abstract

**Background:**

Nursing and health care research are increasingly using e-questionnaires and e-forms for data collection and survey conduction. The main reason lies in costs, time and data-entry errors containment, increased flexibility, functionality and usability. In spite of this growing usage, no specifc and comprehensive guidelines for designing and submitting e-questionnaires have been produced so far.

**Objective:**

The aim of this review is to collect information on the current best practices, taking them from various fields of application. An evaluation of the efficacy of the single indication is provided.

**Method:**

A literature review of guidelines currently available on WebSM (Web Survey Methodology) about electronic questionnaire has been performed. Four search strings were used: “Electronic Questionnaire Design”, “Electronic Questionnaire”, “Online Questionnaire” and “Online survey”. Articles’ inclusion criteria were English language, relevant topic in relation to the aim of the research and the publication date from January 1998 to July 2014.

**Results:**

The review process led to identify 48 studies. The greater part of guidelines is reported for Web, and e-mail questionnaire, while a lack of indications emerges especially for app and e-questionnaires.

**Conclusion:**

Lack of guidelines on e-questionnaires has been found, especially in health care research, increasing the risk of use of ineffective and expensive instruments; more research in this field is needed.

## 1 INTRODUCTION

Technological development and Internet diffusion have changed methods of data collection: World Wide Web and electronic tools represent new challenges for researchers, as these are the newest means to connect people and collect information. These advancements also affect research in clinical settings, where patients and healthcare providers are today more prone to Interent use and more expert on electronic devices like personal computers, tablets, mobile phones, smart phones and others. In nursing research, information is often collected directly from the patient, in order to minimize data entry errors [1, 2]. This method of data collection is called Patient Reported Oucomes PRO (MeSH), Assessment of the quality and effectiveness of health care as measured and directly reported by the patient, and is useful to investigate some health care aspects like patient illness perception, level of pain and efficacy of nurse’s interventions. One of the most important instruments of data collection is the questionnaire, allowing both a self-administration and an operator-assisted interview. Its validity is based on the consistency of gathered information with patients' actual conditions, health perception and thoughts.

Over the last 30 years, traditional survey methods as paper questionnaires or telephone interview, have been partially replaced by new collection instruments designed for electronic tools [3].

Although literature is rich of guidelines on how to create and use paper questionnaires, not all of such indications are applicable to electronic counterparts [1]: for example, while evidence on wording and item definition can be the same for all questionnaires, indications about layout, privacy or question structure may change depending on the type of device. Compared to traditional paper-based methods, electronic questionnaires offer several advantages like cost reduction [4], speed in collection and data analysis [5], personalized design for target sample, lack of influence of researcher presence, comfort for respondents who can complete the questionnaire when and where they prefer, flexibility, functionality, usability, allowing inclusion of pop-up instruction, error messages, link incorporation and making possible to encode difficult skip patterns making such patterns vitually invisible to respondents. However, e-forms present also important disadvantages, able to potentially compromise survey success. Firstly, a sample cannot be representative if the sampling process is not controlled [5], and users can be worried for their privacy or have concerns about data circulation through a network. Furthermore, e-questionnaires can incur in typical errors due to their technological characteristics: e-mail forms can be read as a spam message, or instrument’s format can be incompatible with users’ device.

Literature reports four categories of errors: coverage, non-response, sampling and measurement errors [3, 5, 6]. Coverage error (*i*) occurs when not all members of a population have the same chance to be included in the sample; non response error (*ii*) happens when potential users fail to respond to survey invitation. Sampling errors (*iii*) are a consequence of data collection methods: subjects of interest can be excluded due to technological limitations as low connection, small bandwidth, browser configuration or different devices. Finally, measurement errors (*iv*) occur when ambiguous or incorrect question wording causes imprecise and contradictory answers.

The aim of the study is (*i*) to help researchers in the nursing area in limiting potential survey errors, through a critical review of the main guidelines published over the last decade on electronic questionnaire, and (*ii*) to highlight which aspects of e-forms still need to be analyzed by literature. The study represents a pratical guide to help health researchers and nurses creating an effective e-questionnaire, adapted to the target population and able to collect relevant information.

## 2 METHODS

The present study is a review of the main guidelines currently present in literature about electronic questionnaire, including an overview on efficacy of these recommendations.

Literature review has been conducted using WebSM (Web Survey Methodology), a database specifically dedicated to address the methodological issues of Web surveys. The same search strings have been used to collect review and trial articles. Key words introduced in the advanced search of the website were: *Electronic Questionnaire Design* (search string 1), *Electronic Questionnaire* (search string 2), *Online Questionnaire* (search string 3), *Online survey* (search string 4). Articles’ inclusion criteria were English language, relevant topic in relation to the aim of the research and the publication date from January 1998 to July 2014.

## 3 RESULTS

Fig. (**1**) shows a flow chart of the process which led to identify 18 articles, relevant for the review, out of initial 1,073.

Additional 23 articles found in the references of the 18 selected papers, have been included, for a total of 41 articles. Table (**1**) provides a summary of categories used for classifying e-forms aspects.

Results are organized in 4 different tables: findings from 16 papers were used to create Table (**2**) (Guidelines Table) and Table (**3**) (Guidelines’ Distribution Table), while findings from 25 papers contributed to create Table (**4**) (Efficacy Table). 

Table (**2**) includes guidelines on electronic questionnaire. In this table, indications have been distributed among four types of e-questionnaire:

- simple electronic questionnaire *via* personal computer, with a specific software program designed for the survey [4, 7];
- e-mail questionnaire, a type of e-survey *via* network system in which the questionnaire is sent as an e-mail message [6];
- online questionnaire, directly inserts on the host website, and designed as a web page with a URL [6];
- “App” questionnaire, downloaded as a Smart phone application.

Columns of Table include four types of e-questionnaires, while rows show main categories of guidelines: survey development, questionnaire design, and questionnaire layout and informatics accessibility. The first category, survey development, lists indications on what researcher should do before questionnaire distribution. In the questionnaire design category, we can find evidence about how to profile target audience, ensuring data quality and creating the welcome page, including specific criteria to formulate answers and questions. The row on questionnaire layout provides indications about tool’s visual configuration, with attention to colour use and choice of images. Finally, the category on information accessibility includes guidelines on costs, wording, and indications to ensure an easy and safe utilization of the questionnaire.

From literature review, it is possible to assess that the procedures identified for item’s definition are the same for the different kinds of electronic questionnaire; they are, basically, based on literature review, interviews, focus-group sessions, with potential respondents or experts (Table **2**).

Also, the sample selection procedures are similar, but, specifically, for e-mail questionnaire and web questionnaire, guidelines address the issue of achievement of a desired level of randomness and representativeness of non-probability samples. Moreover, in these cases, it is important to use a database with valid e-mail addresses and permission by potential respondents to send surveys Table (**2**). As to testing and pretesting procedures, the guidelines across different electronic forms are similar, but it is important to take into account that for Web and e-mail questionnaire, virtual pretesting and testing is useful to ensure quick and effective control of the questionnaire (Table **2**).

The validation procedures in term of reliability and validity are identical across different electronic questionnaire forms Table (**2**) and are comparable to procedures provided for paper questionnaire [8]. Shows which aspects of electronic questionnaires have been more investigated, and which ones need further analysis. For example, in the category about survey development Table (**3**), the majority of articles concern e-mail and web questionnaires, while four papers address simple e-forms [1, 9, 10, 14] and only one article discusses app-forms [9].

For what concerns efficacy, Table (**4**) reports estimates of the efficacy of some of the most relevant topics: login procedures, use of reminder letters, progress indicator, questionnaire length and cover messages. As shown in the table, use of personalized invitation [4], adoptions of mixed strategies [9], creation of plain questionnaire, reduction of text length [10], use of reminder letters or telephone contacts [11], inclusion of semiautomatic login procedure [12-14] and adoption of strong privacy policies, are all elements that have the potential to increase response rates. On the other side, including in the cover letter or in the survey introduction an estimate of the completion time does not seem to significantly influence user’s attitudes.

### 4 DISCUSSION

Literature review demonstrated a lack of indications about app and off-line e-questionnaires, while it has reported a wide collection of guidelines on e-mail and web forms. While many indications for online questionnaires can be applied also to simple e-forms, the same does not hold for app versions, since every type of device needs specific guidance in terms of media characteristics and electronic limitations. Colour and graphic use, as well as answers formulation and scrolling choice, can positively or negatively influence response rate: researcher for example should evaluate if scrolling is comfortable for smart phones users, or if a complex configuration does not increase download time. These and other guidelines on questionnaire layout should be taylored depending on type of electronic device. For example, for mail web questionnaire it is important to consider sampling procedures that take into account the representativeness of non-probability samples [5].

Such a lack of evidence affects also the entire process of questionnaire creation. For example, only one article deals with questionnaire design, explaining question types as rank order questions, categorical or nominal questions, magnitude estimate questions, ordinal questions or Likert scale [3, 9].

The category of information accessibility is the most discussed, consistently with the current interest in electronic tools. A large number of studies investigated topics like security (login procedure, privacy protection), strategies to encourage and help users (cover letter, instructions, reminders, users competence), technical procedures to create a simple tool (skip and filter questions, screen and type of device, technical procedures, browser and file size), ways to ensure data quality (repeated answer, confirmation message, thank you page), and indications to create a visually attractive questionnaire, in order to reduce dropout rates and increase users’ responses (progress indicator, one page and multiple page design, wording, phraseology, multiple choice, costs).

Furthermore, while the choice of the topic is relevant in increasing data quality [45], the use of a grid design does not seem to improve it. Respondents’ appreciation on the survey is positively conditioned by the inclusion of progress indicators, as it influences perceptions on questionnaire length, and by the adoption of graphical details that make questionnaire more enjoyable. However, effectiveness of most of the guidelines still remains uncertain: the completion rate is always higher in PC questionnaire compared to app forms, also for sensitive topics; the header image is not affected by “banner blindness” phenomenon and can partially influence user answers [28]; finally, use of progress indicator and first question configuration have little or no effect on dropout and completeness rates [42].

It is important to highlight that the majority of retrieved papers concern marketing and epidemiological topics, while just a few studies have been conducted in clinical setting. Implementation of new electronic tools and adaptation of existing guidelines to the needs of health care and nursing researchers can contribute to reinforce efficacy in this particular setting. This is the case of users with particular needs, for whom literature does not report any specific guideline, but for a few articles [21] giving general information on font size, colours, and button size for subjects with visual or motor difficulties. Due to absence of particular indications for patients with disabilities, the risk to exclude part of the population is still present, with potential, eligible respondents not being able to participate because of their inability to use the instrument.

Lack of guidelines on e-questionnaires also has consequences in health research, especially for nurses (ideal users of this survey tool) [46]. Questionnaires, in nursing research, are useful tools also in simple research studies [47]. For these reasons, the shortage of methodological guidelines concerning the construction, validation and administration of questionnaires, particularly in electronic form, may increase the risk of use of ineffective and expensive instruments to conduct surveys.

In order to facilitate subjects’ participation and collect high-quality data, nurses should consider new technologies as resources to communicate with patients that use electronic tools in daily routine.

## CONCLUSION

Literature review revealed the need of further implementation of guidelines on electronic questionnaires, particularly in nursing and health care research. The current research is suitable to be the starting point of future improvement: future studies on e-questionnaires will update this guidelines collection, including more accurate evidence in line with technological development and population characteristics. Finally, trials on the efficacy of guidelines have to be implemented in order to verify the validity of the indications coming from the current literature. The evolution of electronic techniques for data collection, and their integration into research practice, should always be consistent with the general criteria of validity and reliability, in order to ensure integrity of the scientific process and provide relevant information.

## Figures and Tables

**Fig. (1):**
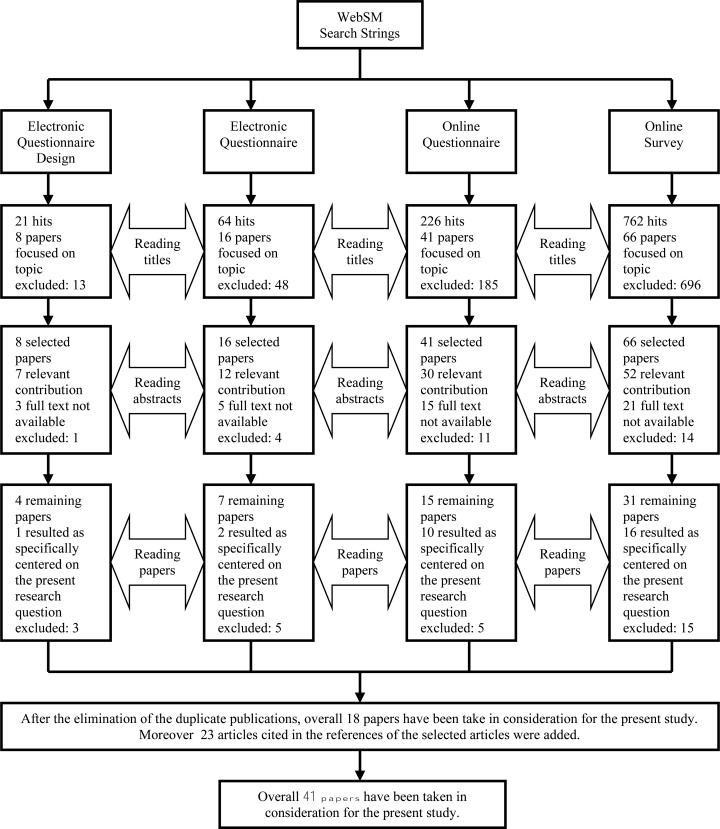
Flow chart of the paper selection process for the review.

**Table 1:** Summary of categories used for classifying e-forms aspects.

**SURVEY’S DEVELOPMENT**	**QUESTIONNAIRE DESIGN**	**QUESTIONNAIRE LAYOUT**	**INFORMATIC ACCESSIBILITY**
**1. Item definition** *Strategies to develop questionnaire’s items* **2. Sample selection** *Type of sample selection, strategies and risk of bias* **3. Training** *How to teach the patient the use of the system* **4. Testing and pretesting** *How to perform tests and pretest to verify the quality, the reliability and the validity of the questionnaire* **5. Reliability test** *How to test questionnaire’s reliability* **6. Validity test** *How to test questionnaire’s validity*	**1. Profile the target audience** *Set the survey topic to the target audience* **2. Quality of data** *Strategies to collect data and implement the response rate* **3. Welcome page** *How to develop the welcome page and what information include in it* **4. Open/closed questions** *When to choose open/closed questions and how to construct them* **5. Rank order questions** *When and how to use rank order questions* **6. Categorical or nominal questions** *When and how to use categorical or nominal questions* **7. Magnitude estimate questions** *When and how to use magnitude estimate questions* **8. Ordinal questions** *When and how to use ordinal questions* **9. Likert scale questions** *When and how to use Likert scale questions* **10. Indeterminate and other options** *When and how to use “indeterminate” and “other” response options* **11. Open/closed response** *How to use open/ closed response*	**1. Questionnaire length** *Indications for questionnaire’s length* **2. Questionnaire structure and questions’ order** *Indications for how to structure the initial question, and how to order the different type of questions* **3. Layout** *General indications for how to organize the visual presentation of the questionnaire* **4. Scrolling** *Indications for how to use and when use the scroll function* **5. Font size and style** *How to choose the type and the size of the font* **6. Color** *How to use colors in questionnaire’s layout* **7. Questions’ graphic** *Indications for the visual appearance of questions* **8. Answers’ graphic** *Indications for visual appearance of responses* **9. Symbols and imagines** *Indications for how to include graphical language in the electronic questionnaire* **10. Tables and frame** *Indications for how to use tables and frame* **11. Visual analogue scale** *Indications for how to design a visual analogue scale* **12. Matrix questions** *Indications for how to use matrix questions* **13. Drop down boxes** *Indications for how to use and design drop down boxes* **14. Radio buttons** *Indications for how to use and design radio buttons* **15. Check button** *General indications for how to design check buttons*	**1. Questionnaire distribution and login procedures** *Indications for how to inform the users about the questionnaire, and* **2. Cover letter** *How to design the cover letter and which information include in it* **3. Thank you page and user’s feedback** *How to conclude a questionnaire, thanking the users and giving them the possibility to send a feedback* **4. Confirmation message** *How to include confirmation message, in order to give a feedback to user’s actions* **5. Timeout** *Indications to the use of timeout* **6. Progress indicators** *Indications for the use of progress indicators and other flow indicators to facilitate the questionnaire’s compilation* **7. One page design and multiple page design** *Pro and cons of the chosen design* **8. Phraseology** *Indications on grammar and content in the formulation of questions* **9. Language and wording** *Indications for language and how to speak gentle to the users, encouraging them to complete the questionnaire* **10. Multiple choice** *Indications for how to use and design multiple choice answers* **11. Skip/filter question** *Indications for how to use and how to design filter questions, in order to facilitate questionnaire flow* **12. Invitation and reminders** *Indications for the use of reminders and invitations to encourage the users to respond* **13. Instructions** *Indications for how to present instructions to complete the questionnaire* **14. Anonymity and privacy** *Indications to manage and ensure users’ anonymity and privacy* **15. Costs** *Costs that sometimes the users have to pay to participate at the survey* **16. Repeated answers** *How to use email address, cookies, IP number and thank you message to avoid repeated answers* **17. Screen and type of devices** *Indications for screen size and questionnaire’s adaptation to the type of device* **18. User’s computer competence** *How to design the system taking in account the sills of potential users* **19. Technical procedures** *Which technical procedures can be requested to the users in order to participate at the survey* **20. Browser and file size** *Technical indications for browser use and files’ size*

**Table 2:** Guidelines on questionnaire design and implementation.

**First ** **category**	**Second Category**	**Electronic Questionnaire**	**Email Questionnaire**	**Web Questionnaire**	**App Questionnaire**
**Survey’s Development**	*Item* *definition*	Generate items through literature review, interviews, focus-group sessions, with potential respondents or experts. [3, 9] Create a “table of specification”, to ensure that sufficient items have been generated. [9] Develop the study to further implement best practices. [9] Insert items consistent with the aim of the survey. Unnecessary items can affect data quality.	Generate items through literature review, interviews, focus-group sessions, with potential respondents or experts. [10] Create a “table of specification”, to ensure that sufficient items have been generated. [9] Develop the study to further implement best practices. [9] Insert items consistent to the aim of the survey. Unnecessary items can affect data quality.	Generate items through literature review, interviews, focus-group sessions, with potential respondents or experts. [10] Create a “table of specification”, to ensure that sufficient items have been generated. [9] Develop the study to further implement best practices. [9] Insert items consistent to the aim of the survey. Unnecessary items can affect data quality.	Generate items through literature review, interviews, focus-group sessions, with potential respondents or experts. [10] Create a “table of specification”, to ensure that sufficient items have been generated. [9] Develop the study to further implement best practices. [9] Insert items consistent to the aim of the survey. Unnecessary items can affect data quality.
	*Sample selection*	To not have a self-selected group, non-probability sampling should meet the condition of sufficient response rate. [10] Direct effort at decreasing the occurrence of sampling measurement and non-response errors. [5] Bias must be avoided, both in the selection of the population being studied and in the responses made. [5] Use online panels because quotas and screening can help to target the proper respondents in a demographically balanced manner. [11]	To not have a self-selected group, non-probability sampling should meet the condition of sufficient response rate. [1] These procedures should be considered for achieving the desired level of randomness and representativeness of non-probability samples. • Random selection of email addresses from newsgroup • Use of stratified random samples of proprietary bulletin board users • Employment of a sampling frame from list of users who have free access to internet • Use of a stratified sample of individuals whose email-addresses are obtained from Usenet newsgroup. [5] Direct effort at decreasing the occurrence of sampling measurement and non-response errors. [5] Bias must be avoided, both in the selection of the population being studied and in the responses made. [5] Used web surveys when wide geographic coverage is sought. [11] Have a database with valid e-mail addresses and permission by potential respondents to send surveys. [1] Use online panels because quotas and screening can help to target the proper respondents in a demographically balanced manner. [1]	To not have a self-selected group, non-probability sampling should meet the condition of sufficient response rate. [1] These procedures should be considered for achieving the desired level of randomness and representativeness of non-probability samples. • Random selection of email addresses from newsgroup • Use of stratified random samples of proprietary bulletin board users • Employment of a sampling frame from list of users who have free access to internet • Use of a stratified sample of individuals whose email-addresses are obtained from Usenet newsgroup. [5] Direct effort at decreasing the occurrence of sampling measurement and non-response errors. [5] Bias must be avoided, both in the selection of the population being studied and in the responses made. [5] Used web surveys when wide geographic coverage is sought. [11] Have a database with valid e-mail addresses and permission by potential respondents to send surveys. [1] Use online panels because quotas and screening can help to target the proper respondents in a demographically balanced manner. [1]	To not have a self-selected group, non-probability sampling should meet the condition of sufficient response rate. [1] Direct effort at decreasing the occurrence of sampling measurement and non-response errors. [5] Bias must be avoided, both in the selection of the population being studied and in the responses made. [5] Used web surveys when wide geographic coverage is sought. [11] Use online panels because quotas and screening can help to target the proper respondents in a demographically balanced manner. [1]
	*Training*	Consider the idea to make a short period of training in order to make patients capable of using the system. [1]			
	*Testing and pretesting*	Perform tests to verify the quality of the questionnaire: • Pre testing-to ensure a clear interpretation of individual questions • Pilot testing-to review the relevance, flow, arrangement and wording of the questionnaire • Clinical sensibility testing-to ensure the adherence of the questionnaire to the survey objective. [11] Development plan and acceptance testing must test the suitability of the application. [9] Do not test every aspect of the application, simplify the testing requirement using design based on principles have been tried in similar context in the past. [11] The competence test must verify technical and executive capabilities, imperfections and answer problems of the questionnaire. [11] Test the questionnaire using monitors with different resolutions in order to ensure the comprehensibility. [6] Sometimes is better to repeat questions slightly reworded in order to assess consistency of response. [6] Questionnaire pre-test is useful if it is done with multiple browsers and screen settings in order to ensure their applicability and accessibility. [12] Pre-test is useful to reduce the problem of unclear answering instructions; it is recommended the use of opt-in popup windows that can be accessed if a question is not understood. [1] Set the tab order on the pages to ensure the that text is readable in a logical way. [1] Run the pre-test on various members of the target group or on an experts group. [13]	Perform tests to verify the quality of the questionnaire: • Pre testing-to ensure a clear interpretation of individual questions • Pilot testing-to review the relevance, flow, arrangement and wording of the questionnaire • Clinical sensibility testing-to ensure the adherence of the questionnaire to the survey objective. [14] Development plan and acceptance testing must test the suitability of the application. [9] Do not test every aspect of the application, simplify the testing requirement using design based on principles have been tried in similar context in the past. [11] The competence test must verify technical and executive capabilities, imperfections and answer problems of the questionnaire. [11] Ensure that the arrangement and formation of sent file do not change after receiving or responding the email questionnaire. [6] Test the questionnaire using several Web explorers and monitors with different resolutions in order to ensure the comprehensibility. [6] Sometimes is better to repeat questions slightly reworded in order to assess consistency of response. [6] Questionnaire pre-test is useful if it is done with multiple browsers and screen settings in order to ensure their applicability and accessibility. [12] Pre-test is useful to reduce the problem of unclear answering instructions; it is recommended the use of opt-in popup windows that can be accessed if a question is not understood. [1] Set the tab order on the pages to ensure the that text is readable in a logical way. [1] Run the pre-test on various members of the target group or on an experts group. [13] For Web and e-mail questionnaire, virtual pretesting and testing is useful to ensure quick and effective control of the questionnaire. [14]	Perform tests to verify the quality of the questionnaire: • Pre testing-to ensure a clear interpretation of individual questions • Pilot testing-to review the relevance, flow, arrangement and wording of the questionnaire • Clinical sensibility testing-to ensure the adherence of the questionnaire to the survey objective. [14] Development plan and acceptance testing must test the suitability of the application. [9] Do not test every aspect of the application, simplify the testing requirement using design based on principles have been tried in similar context in the past. [11] The competence test must verify technical and executive capabilities, imperfections and answer problems of the questionnaire. [11] Test the questionnaire using several Web explorers and monitors with different resolutions in order to ensure the comprehensibility. [6] Sometimes is better to repeat questions slightly reworded in order to assess consistency of response. [6] Questionnaire pre-test is useful if it is done with multiple browsers and screen settings in order to ensure their applicability and accessibility. [12] Pre-test is useful to reduce the problem of unclear answering instructions; it is recommended the use of opt-in popup windows that can be accessed if a question is not understood. [1] Set the tab order on the pages to ensure the that text is readable in a logical way. [1] Run the pre-test on various members of the target group or on an experts group. [13] For Web and e-mail questionnaire, virtual pretesting and testing is useful to ensure quick and effective control of the questionnaire. [14]	Perform tests to verify the quality of the questionnaire: • Pre testing-to ensure a clear interpretation of individual questions • Pilot testing-to review the relevance, flow, arrangement and wording of the questionnaire • Clinical sensibility testing-to ensure the adherence of the questionnaire to the survey objective. [14] Development plan and acceptance testing must test the suitability of the application. [9] Do not test every aspect of the application, simplify the testing requirement using design based on principles have been tried in similar context in the past. [11] The competence test must verify technical and executive capabilities, imperfections and answer problems of the questionnaire. [11] Test the questionnaire using several Web explorers and monitors with different resolutions in order to ensure the comprehensibility. [6] Sometimes is better to repeat questions slightly reworded in order to assess consistency of response. [6] Questionnaire pre-test is useful if it is done with multiple browsers and screen settings in order to ensure their applicability and accessibility. [12] Pre-test is useful to reduce the problem of unclear answering instructions; it is recommended the use of opt-in popup windows that can be accessed if a question is not understood. [1] Set the tab order on the pages to ensure the that text is readable in a logical way. [1] Run the pre-test on various members of the target group or on an experts group. [13] For Web and e-mail questionnaire, virtual pretesting and testing is useful to ensure quick and effective control of the questionnaire. [14]
	*Reliability test*	Run a test-retest reliability whether the same question posed to the same individual yields consistent results at different times. [14] Ensure the interrater reliability if you expect different respondents provide similar responses. [9] Ensure the internal consistency, verifying that different items tapping into the same construct are correlated. [9] The reliability assessment must depend on the objective of the survey and the type of data collected. [9]	Run a test-retest reliability whether the same question posed to the same individual yields consistent results at different times. [9] Ensure the interrater reliability if you expect different respondents provide similar responses. [9] Ensure the internal consistency, verifying that different items tapping into the same construct are correlated. [9] The reliability assessment must depend on the objective of the survey and the type of data collected. [9]	Run a test-retest reliability whether the same question posed to the same individual yields consistent results at different times. [9] Ensure the interrater reliability if you expect different respondents provide similar responses. [9] Ensure the internal consistency, verifying that different items tapping into the same construct are correlated. [9] The reliability assessment must depend on the objective of the survey and the type of data collected. [9]	Run a test-retest reliability whether the same question posed to the same individual yields consistent results at different times. [9] Ensure the interrater reliability if you expect different respondents provide similar responses. [9] Ensure the internal consistency, verifying that different items tapping into the same construct are correlated. [9] The reliability assessment must depend on the objective of the survey and the type of data collected. [9]
	*Validity test*	Ensure face validity, testing whether the questionnaire measures what it intends to measure during clinical sensibility testing. [9] Ensure content validity, verifying if questionnaire content accurately assess all fundamental aspects of the topic. [9] Ensure construct validity if it is not possible to identify specific criteria that adequately define the construct being measured. [9] Define criterion validity, comparing questionnaire responses to a “gold standard”. [9] Engage investigators in one or more assessments of instrument validity, depending on current and anticipated uses of the questionnaire. [9]	Ensure face validity, testing whether the questionnaire measures what it intends to measure during clinical sensibility testing. [9] Ensure content validity, verifying if questionnaire content accurately assess all fundamental aspects of the topic. [9] Ensure construct validity if it is not possible to identify specific criteria that adequately define the construct being measured. [9] Define criterion validity, comparing questionnaire responses to a “gold standard”. [9] Engage investigators in one or more assessments of instrument validity, depending on current and anticipated uses of the questionnaire. [9]	Ensure face validity, testing whether the questionnaire measures what it intends to measure during clinical sensibility testing. [9] Ensure content validity, verifying if questionnaire content accurately assess all fundamental aspects of the topic. [9] Ensure construct validity if it is not possible to identify specific criteria that adequately define the construct being measured. [9] Define criterion validity, comparing questionnaire responses to a “gold standard”. [9] Engage investigators in one or more assessments of instrument validity, depending on current and anticipated uses of the questionnaire. [9]	Ensure face validity, testing whether the questionnaire measures what it intends to measure during clinical sensibility testing. [9] Ensure content validity, verifying if questionnaire content accurately assess all fundamental aspects of the topic. [9] Ensure construct validity if it is not possible to identify specific criteria that adequately define the construct being measured. [9] Define criterion validity, comparing questionnaire responses to a “gold standard”. [9] Engage investigators in one or more assessments of instrument validity, depending on current and anticipated uses of the questionnaire. [9]
**QUESTIONNAIRE DESIGN**	*Profile the target audience*	Tailor a questionnaire to the interests and style of the target audience. [9] The survey topic must be relevant to the target group. [5] Design the questionnaire in order to give the user the impression of participating in an interesting conversation. [5] Focus on in-the-moment data, because increases interest in the survey. [14] Adapt the survey protocols according legal and ethical considerations of the specific country and culture.	Tailor a questionnaire to the interests and style of the target audience. [15] The survey topic must be relevant to the target group. [5] Design the questionnaire in order to give the user the impression of participating in an interesting conversation. [5] Focus on in-the-moment data, because increases interest in the survey. [14] Adapt the survey protocols according legal and ethical considerations of the specific country and culture.	Tailor a questionnaire to the interests and style of the target audience. [15] The survey topic must be relevant to the target group. [5] Design the questionnaire in order to give the user the impression of participating in an interesting conversation. [5] Focus on in-the-moment data, because increases interest in the survey. [14] Adapt the survey protocols according legal and ethical considerations of the specific country and culture.	Tailor a questionnaire to the interests and style of the target audience. [15] The survey topic must be relevant to the target group. [5] Design the questionnaire in order to give the user the impression of participating in an interesting conversation. [5] Focus on in-the-moment data, because increases interest in the survey. [14] Adapt the survey protocols according legal and ethical considerations of the specific country and culture.
	*Quality of data*	Consider to collaborate with a biostatistician to ensure that data required for analyses are obtained in a usable format. [15] Consider that the collation of the data can be problematic, because consistent structure within responses can only be suggested, not enforced. [9] It is possible increase response rate and accuracy of responses by paying attention to access problems, the motivation and cognition of respondents. [5] Consider these sources’ effects: no interviewer, respondent control, visual processing of information. [5] Past research can be useful in order to develop an implementation strategy to improving respondent to self-administered questionnaires. [16] Analyze the phenomenon of non-response and it is possible causes: technological considerations, questionnaire design decisions, respondent computer skills, as well as implementation decisions. [16] In data collection do not consider default values, namely common or sensible values that are displayed before the user enters a value, and which are taken as data when the user does not make a specific choice. Responses should always result from an action by the user. [16] Improve the response rate creating a short and relevant survey, based on the interest of the target population. [11] Consider that the decision to participate at the questionnaire is influenced also by the flexibility of response time. [1] Ensure that the experience is comparable across different regions, by creating a survey protocol applicable in different context. [15]	Consider to collaborate with a biostatistician to ensure that data required for analyses are obtained in a usable format. [10] Consider that the collation of the data can be problematic, because consistent structure within responses can only be suggested, not enforced. [9] It is possible increase response rate and accuracy of responses by paying attention to access problems, the motivation and cognition of respondents. [5] Use properly the subject line of email messages in order to inform potential respondents of the study. [5] Consider these sources’ effects: no interviewer, respondent control, visual processing of information. [5] Past research can be useful in order to develop an implementation strategy to improving respondent to self-administered questionnaires. [16] Analyze the phenomenon of non-response and it is possible causes: technological considerations, questionnaire design decisions, respondent computer skills, as well as implementation decisions. [16] In data collection do not consider default values, namely common or sensible values that are displayed before the user enters a value, and which are taken as data when the user does not make a specific choice. Responses should always result from an action by the user. [16] Improve the response rate creating a short and relevant survey, based on the interest of the target population. [11] Consider that the decision to participate at the questionnaire is influenced also by the flexibility of response time. [1] Ensure that the experience is comparable across different regions, by creating a survey protocol applicable in different context. [15]	Consider to collaborate with a biostatistician to ensure that data required for analyses are obtained in a usable format. [10] Consider that the collation of the data can be problematic, because consistent structure within responses can only be suggested, not enforced. [9] It is possible increase response rate and accuracy of responses by paying attention to access problems, the motivation and cognition of respondents. [5] Use properly the subject line of email messages in order to inform potential respondents of the study. [5] Consider these sources’ effects: no interviewer, respondent control, visual processing of information. [5] Past research can be useful in order to develop an implementation strategy to improving respondent to self-administered questionnaires. [16] Analyze the phenomenon of non-response and it is possible causes: technological considerations, questionnaire design decisions, respondent computer skills, as well as implementation decisions. [16] In data collection do not consider default values, namely common or sensible values that are displayed before the user enters a value, and which are taken as data when the user does not make a specific choice. Responses should always result from an action by the user. [16] Improve the response rate creating a short and relevant survey, based on the interest of the target population. [11] Consider that the decision to participate at the questionnaire is influenced also by the flexibility of response time. [1] Ensure that the experience is comparable across different regions, by creating a survey protocol applicable in different context. [15]	Consider to collaborate with a biostatistician to ensure that data required for analyses are obtained in a usable format. [10] Consider that the collation of the data can be problematic, because consistent structure within responses can only be suggested, not enforced. [9] It is possible increase response rate and accuracy of responses by paying attention to access problems, the motivation and cognition of respondents. [5] Consider these sources’ effects: no interviewer, respondent control, visual processing of information. [5] Past research can be useful in order to develop an implementation strategy to improving respondent to self-administered questionnaires. [16] Analyze the phenomenon of non-response and it is possible causes: technological considerations, questionnaire design decisions, respondent computer skills, as well as implementation decisions. [16] In data collection do not consider default values, namely common or sensible values that are displayed before the user enters a value, and which are taken as data when the user does not make a specific choice. Responses should always result from an action by the user. [16] Improve the response rate creating a short and relevant survey, based on the interest of the target population. [11] Consider that the decision to participate at the questionnaire is influenced also by the flexibility of response time. [1] Ensure that the experience is comparable across different regions, by creating a survey protocol applicable in different context. [15] Ensure that online data is comparable with mobile data, despite the different mode of data collection. [10]
	*Welcome page*	Signatures of departmental stationery should be present. [10] Affirm that the recipient’s participation is imperative to the success of the survey. [9] Make sure that welcome page and first questions are relevant, fast, and easy. [9] Introduce the questionnaire with a welcome screen to motivate users, to emphasize the ease of responding and to teach the respondent how to proceed. [16] Include a short and understandable introduction to explain the topic of the survey. [12] Try to persuade participants to agree to be surveyed and to share personal data. [12] Explain clearly the aim of the survey. [1] Use the questionnaire’s introduction to capture the users’ interest. [14]	Signatures of departmental stationery should be present. [14] Affirm that the recipient’s participation is imperative to the success of the survey. [9] Make sure that welcome page and first questions are relevant, fast, and easy. [9] Introduce the questionnaire with a welcome screen to motivate users, to emphasize the ease of responding and to teach the respondent how to proceed. [16] Include a short and understandable introduction to explain the topic of the survey. [12] Try to persuade participants to agree to be surveyed and to share personal data. [12] Explain clearly the aim of the survey. [1] Use the questionnaire’s introduction to capture the users’ interest. [14]	Signatures of departmental stationery should be present. [14] Affirm that the recipient’s participation is imperative to the success of the survey. [9] Make sure that welcome page and first questions are relevant, fast, and easy. [9] Introduce the questionnaire with a welcome screen to motivate users, to emphasize the ease of responding and to teach the respondent how to proceed. [16] Include a short and understandable introduction to explain the topic of the survey. [12] Include a site map to help the users during the navigation. [12] Try to persuade participants to agree to be surveyed and to share personal data. [12] Explain clearly the aim of the survey. [1] Use the questionnaire’s introduction to capture the users’ interest. [14]	Signatures of departmental stationery should be present. [14] Affirm that the recipient’s participation is imperative to the success of the survey. [9] Make sure that welcome page and first questions are relevant, fast, and easy. [9] Introduce the questionnaire with a welcome screen to motivate users, to emphasize the ease of responding and to teach the respondent how to proceed. [16] Include a short and understandable introduction to explain the topic of the survey. [12] Include a site map to help the users during the navigation. [12] Try to persuade participants to agree to be surveyed and to share personal data. [12] Explain clearly the aim of the survey. [1] Use the questionnaire’s introduction to capture the users’ interest. [14]
	*Open/ closed questions*	Use open-ended questions if there are too many possible response options or when the response possibilities are unknown at the time of administering the questionnaire. [14] Use open-ended questions to not impose response categories onto the respondents. [12] Use open-ended questions with sparingly, because of the complexity of the analysis. [12] Use closed-ended questions when the goal is to obtain data that is ranked or rated and when the ratings’ order is know before. [12] Use closed-ended questions if statistical data is required and where is better to count the number of choices rather than analyze free text. [12] Closed-ended questions should be completeness, exclusivity and clarity. [12] In closed question, answer’s categories must be complete and should not overlap. [2]	Do not be reluctant to incorporate open ended items in the e-mail questionnaire. [14] Use open-ended questions if there are too many possible response options or when the response possibilities are unknown at the time of administering the questionnaire. [5] Use open-ended questions to not impose response categories onto the respondents. [12] Use open-ended questions with sparingly, because of the complexity of the analysis. [12] Use closed-ended questions when the goal is to obtain data that is ranked or rated and when the ratings’ order is know before. [12] Use closed-ended questions if statistical data is required and where is better to count the number of choices rather than analyze free text. [12] Closed-ended questions should be completeness, exclusivity and clarity. [12] In closed question, answer’s categories must be complete and should not overlap. [2]	Use open-ended questions if there are too many possible response options or when the response possibilities are unknown at the time of administering the questionnaire. [14] Use open-ended questions to not impose response categories onto the respondents. [12] Use open-ended questions with sparingly, because of the complexity of the analysis. [12] Use closed-ended questions when the goal is to obtain data that is ranked or rated and when the ratings’ order is know before. [12] Use closed-ended questions if statistical data is required and where is better to count the number of choices rather than analyze free text. [12] Closed-ended questions should be completeness, exclusivity and clarity. [12] In closed question, answer’s categories must be complete and should not overlap. [2]	Use open-ended questions if there are too many possible response options or when the response possibilities are unknown at the time of administering the questionnaire. [14] Use open-ended questions to not impose response categories onto the respondents. [12] Use open-ended questions with sparingly, because of the complexity of the analysis. [12] Use closed-ended questions when the goal is to obtain data that is ranked or rated and when the ratings’ order is know before. [12] Use closed-ended questions if statistical data is required and where is better to count the number of choices rather than analyze free text. [12] Closed-ended questions should be completeness, exclusivity and clarity. [12] In closed question, answer’s categories must be complete and should not overlap. [2] Prefer closed questions for app surveys. [14] Tailor the question types to the specific design constraints and strengths of the mobile devices. [15]
	*Rank order questions*	Rank order question can be used to require the user to rank items in a list according to some criteria. [10]	Rank order question can be used to require the user to rank items in a list according to some criteria. [12]	Rank order question can be used to require the user to rank items in a list according to some criteria. [12]	Rank order question can be used to require the user to rank items in a list according to some criteria. [12]
	*Categorical or nominal questions*	Categorical or nominal question can be used to require the user to chose one or more from a selection of categories listed as the response options. [12] Categories listed must be all inclusive and exhaustive. [12] Use this type of questions to obtain sensitive information. [12]	Categorical or nominal question can be used to require the user to chose one or more from a selection of categories listed as the response options. [12] Categories listed must be all inclusive and exhaustive. [12] Use this type of questions to obtain sensitive information. [12]	Categorical or nominal question can be used to require the user to chose one or more from a selection of categories listed as the response options. [12] Categories listed must be all inclusive and exhaustive. [12] Use this type of questions to obtain sensitive information. [12]	Categorical or nominal question can be used to require the user to chose one or more from a selection of categories listed as the response options. [12] Categories listed must be all inclusive and exhaustive. [12] Use this type of questions to obtain sensitive information. [12]
	*Magnitude estimate questions*	Magnitude estimate questions can be used to require comparative judgments. [12]	Magnitude estimate questions can be used to require comparative judgments. [12]	Magnitude estimate questions can be used to require comparative judgments. [12]	Magnitude estimate questions can be used to require comparative judgments. [12]
	*Ordinal questions*	Ordinal questions can be used only when the topic is well-defined and the response options represent a gradation along a single dimension. [12]	Ordinal questions can be used only when the topic is well-defined and the response options represent a gradation along a single dimension. [12]	Ordinal questions can be used only when the topic is well-defined and the response options represent a gradation along a single dimension. [12]	Ordinal questions can be used only when the topic is well-defined and the response options represent a gradation along a single dimension. [12]
	*Likert scale questions*	Likert scale questions can be used to require users to indicate their level of agreement with a particular statement. [12] Provide brief instruction per question to explain how answers should be provided. [12] Consider that the use of intermediate values of response can influence the choice of the user: patients are inclined to choose more neutral values.	Likert scale questions can be used to require users to indicate their level of agreement with a particular statement. [12] Provide brief instruction per question to explain how answers should be provided. [12] Consider that the use of intermediate values of response can influence the choice of the user: patients are inclined to choose more neutral values.	Likert scale questions can be used to require users to indicate their level of agreement with a particular statement. [12] Provide brief instruction per question to explain how answers should be provided. [12] Consider that the use of intermediate values of response can influence the choice of the user: patients are inclined to choose more neutral values.	Likert scale questions can be used to require users to indicate their level of agreement with a particular statement. [12] Provide brief instruction per question to explain how answers should be provided. [12] Consider that the use of intermediate values of response can influence the choice of the user: patients are inclined to choose more neutral values.
	*Indeterminate and other response options*	Include indeterminate or other response options, to avoid “floor and ceiling” effects. [12] Indeterminate response options can be used when binary responses are sought or when respondent knowledge is being probed. [9] Remove questions that demonstrate floor or ceiling effects, or use another response format to increase the range of responses. [9] Use “other” response options to enhance response rates in self administered questionnaires, or use them during questionnaire testing, in order to identify new issues or to elaborate on closed response formats. [9] Include a “do not know” and a “no opinion” response choice in knowledge questions. [9]	Include indeterminate or other response options, to avoid “floor and ceiling” effects. [12] Indeterminate response options can be used when binary responses are sought or when respondent knowledge is being probed. [9] Remove questions that demonstrate floor or ceiling effects, or use another response format to increase the range of responses. [9] Use “other” response options to enhance response rates in self administered questionnaires, or use them during questionnaire testing, in order to identify new issues or to elaborate on closed response formats. [9] Include a “do not know” and a “no opinion” response choice in knowledge questions. [9]	Include indeterminate or other response options, to avoid “floor and ceiling” effects. [12] Indeterminate response options can be used when binary responses are sought or when respondent knowledge is being probed. [9] Remove questions that demonstrate floor or ceiling effects, or use another response format to increase the range of responses. [9] Use “other” response options to enhance response rates in self administered questionnaires, or use them during questionnaire testing, in order to identify new issues or to elaborate on closed response formats. [9] Include a “do not know” and a “no opinion” response choice in knowledge questions. [9]	Include indeterminate or other response options, to avoid “floor and ceiling” effects. [12] Indeterminate response options can be used when binary responses are sought or when respondent knowledge is being probed. [9] Remove questions that demonstrate floor or ceiling effects, or use another response format to increase the range of responses. [9] Use “other” response options to enhance response rates in self administered questionnaires, or use them during questionnaire testing, in order to identify new issues or to elaborate on closed response formats. [9] Include a “do not know” and a “no opinion” response choice in knowledge questions. [9]
	*Open/ closed responses*	Use succinct and unbiased response formats, either “open” or “closed”. [12]	Use succinct and unbiased response formats, either “open” or “closed”. [9]	Use succinct and unbiased response formats, either “open” or “closed”. [9]	Use succinct and unbiased response formats, either “open” or “closed”. [9]
**QUESTIONNAIRE LAYOUT**	*Questionnaire length*	Pay attention to the length of the questionnaire: in general, questions are addressed with 25 or fewer items and at least 5 items in each domain. [9] The length of a questionnaire influences the response rate, because the questionnaire can take up the space of several computer screens. [9] When create a voluntary survey, be careful to keep the questionnaire short. [5] Keep just a few questions in each page. [16] Do not exceed the number of 60 questions in the entire questionnaire. [6] Do not exceed the number of 15-20 questions in the entire questionnaire. Use longer questionnaire only if the users are adequately prepared via a pre notification letter and it is sure that they are interested in the topic and approve of the study's aims. [12] Include only the most essential and relevant questions for the survey. [14] Be careful to the length of the questionnaire, including a small number of short, well focused, understandable questions, avoiding to require excessive concentration. [14] Specific the number of questions included in the questionnaire, in order to reduce the likelihood to abandonment. [17] Keep survey length short, in order to avoid that users speed through the survey to completion. [18]	Pay attention to the length of the questionnaire: in general, questions are addressed with 25 or fewer items and at least 5 items in each domain. [19] The length of a questionnaire influences the response rate, because the questionnaire can take up the space of several computer screens. [9] When create a voluntary survey, be careful to keep the questionnaire short. [5] Keep just a few questions in each page. [16] Do not exceed the number of 60 questions in the entire questionnaire. [6] Do not exceed the number of 15-20 questions in the entire questionnaire. Use longer questionnaire only if the users are adequately prepared via a pre notification letter and it is sure that they are interested in the topic and approve of the study's aims. [12] Include only the most essential and relevant questions for the survey. [14] Be careful to the length of the questionnaire, including a small number of short, well focused, understandable questions, avoiding to require excessive concentration. [14] Specific the number of questions included in the questionnaire, in order to reduce the likelihood to abandonment. [17] Keep survey length short, in order to avoid that users speed through the survey to completion. [18]	Pay attention to the length of the questionnaire: in general, questions are addressed with 25 or fewer items and at least 5 items in each domain. [19] The length of a questionnaire influences the response rate, because the questionnaire can take up the space of several computer screens. [9] The length of a questionnaire must change according to the type of questionnaire: an email questionnaire can be excessively long when placed on a Web site. [5] When create a voluntary survey, be careful to keep the questionnaire short. [5] Keep just a few questions in each page. [16] Do not exceed the number of 60 questions in the entire questionnaire. [6] Do not exceed the number of 15-20 questions in the entire questionnaire. Use longer questionnaire only if the users are adequately prepared via a pre notification letter and it is sure that they are interested in the topic and approve of the study's aims. [12] Include only the most essential and relevant questions for the survey. [14] Be careful to the length of the questionnaire, including a small number of short, well focused, understandable questions, avoiding to require excessive concentration. [14] Specific the number of questions included in the questionnaire, in order to reduce the likelihood to abandonment. [17] Keep survey length short, in order to avoid that users speed through the survey to completion. [18]	Pay attention to the length of the questionnaire: in general, questions are addressed with 25 or fewer items and at least 5 items in each domain. [19] The length of a questionnaire influences the response rate, because the questionnaire can take up the space of several device screens. [9] When create a voluntary survey, be careful to keep the questionnaire short. [5] Keep just a few questions in each page. [16] Do not exceed the number of 60 questions in the entire questionnaire. [6] Do not exceed the number of 15-20 questions in the entire questionnaire. Use longer questionnaire only if the users are adequately prepared via a pre notification letter and it is sure that they are interested in the topic and approve of the study's aims. [12] Include only the most essential and relevant questions for the survey. [14] Be careful to the length of the questionnaire, including a small number of short, well focused, understandable questions, avoiding to require excessive concentration. [14] Specific the number of questions included in the questionnaire, in order to reduce the likelihood to abandonment. [17] Keep survey length short, especially in mobile research, in order to avoid that users speed through the survey to completion. [18]
	*Questionnaire structure and questions’ order*	At first, include simple questions or demographic questions. [19] Include sensitive questions at the end of the questionnaire. [9] Make sure that the initial questions is routine and easy-to-answer questions in order to arouse user’s interest. [9] At the first question include an item that is likely to be interesting, easy to answer, and fully visible on the first screen of the questionnaire. [12] Number consecutively and organize questions according on content and structure. [14] Organize the questionnaire in order to facilitate questionnaire flow. [9, 16] Ask one question at time. [9] Use an item-in-a-series format to place items with the same response categories. [16] Include useful graphical languages to facilitate user’s navigation through the desired response path. [16] Present and organize the questionnaire so that it is attractive and easy to complete, without including too much information for page and align elements horizontally or vertically in order to facilitate the reading. [16] Include delicate, tricky and the most important questions at 1/3 of the questionnaire, when the user has already start to complete it, but he’s not yet bored. [12] Include open-ended questions at the 2/3 of the questionnaire, in order to maintain the users’ interest. [12] If the topic is the same, include open-ended questions before closed-ended questions, to avoid influencing respondents with the fixed option choices. [12] Do not separate a question from its response set. [12] Avoid to require that a user provide an answer to one question before moving on to the next question. [12] In order to reduce survey bias, directs users’ navigation, requiring them to answer questions in the order intended by the study designer. [12] The initial question must be fully visible on the first screen of the questionnaire, interest-getting, easily comprehended and answered by all respondents. Do not use drop-down box or require scrolling in order to see the entire first question. [12]	At first, include simple questions or demographic questions. [20] Include sensitive questions at the end of the questionnaire. [9] Make sure that the initial questions is routine and easy-to-answer questions in order to arouse user’s interest. [9] At the first question include an item that is likely to be interesting, easy to answer, and fully visible on the first screen of the questionnaire. [12] Number consecutively and organize questions according on content and structure. [14] Organize the questionnaire in order to facilitate questionnaire flow. [9, 16] Ask one question at time. [9] Use an item-in-a-series format to place items with the same response categories. [16] Include useful graphical languages to facilitate user’s navigation through the desired response path. [16] Present and organize the questionnaire so that it is attractive and easy to complete, without including too much information for page and align elements horizontally or vertically in order to facilitate the reading. [16] Include delicate, tricky and the most important questions at 1/3 of the questionnaire, when the user has already start to complete it, but he’s not yet bored. [12] Include open-ended questions at the 2/3 of the questionnaire, in order to maintain the users’ interest. [12] If the topic is the same, include open-ended questions before closed-ended questions, to avoid influencing respondents with the fixed option choices. [12] Do not separate a question from its response set. [12] Avoid to require that a user provide an answer to one question before moving on to the next question. [12] In order to reduce survey bias, directs users’ navigation, requiring them to answer questions in the order intended by the study designer. [12] The initial question must be fully visible on the first screen of the questionnaire, interest-getting, easily comprehended and answered by all respondents. Do not use drop-down box or require scrolling in order to see the entire first question. [12]	At first, include simple questions or demographic questions. [20] Include sensitive questions at the end of the questionnaire. [9] Make sure that the initial questions is routine and easy-to-answer questions in order to arouse user’s interest. [9] At the first question include an item that is likely to be interesting, easy to answer, and fully visible on the first screen of the questionnaire. [12] Number consecutively and organize questions according on content and structure. [14] Organize the questionnaire in order to facilitate questionnaire flow. [9, 16] Ask one question at time. [9] Use an item-in-a-series format to place items with the same response categories. [16] Include useful graphical languages to facilitate user’s navigation through the desired response path. [16] Present and organize the questionnaire so that it is attractive and easy to complete, without including too much information for page and align elements horizontally or vertically in order to facilitate the reading. [16] Include delicate, tricky and the most important questions at 1/3 of the questionnaire, when the user has already start to complete it, but he’s not yet bored. [12] Include open-ended questions at the 2/3 of the questionnaire, in order to maintain the users’ interest. [12] If the topic is the same, include open-ended questions before closed-ended questions, to avoid influencing respondents with the fixed option choices. [12] Do not separate a question from its response set. [12] Avoid to require that a user provide an answer to one question before moving on to the next question. [12] In order to reduce survey bias, directs users’ navigation, requiring them to answer questions in the order intended by the study designer. [12] The initial question must be fully visible on the first screen of the questionnaire, interest-getting, easily comprehended and answered by all respondents. Do not use drop-down box or require scrolling in order to see the entire first question. [12]	At first, include simple questions or demographic questions. [20] Include sensitive questions at the end of the questionnaire. [9] Make sure that the initial questions is routine and easy-to-answer questions in order to arouse user’s interest. [9] At the first question include an item that is likely to be interesting, easy to answer, and fully visible on the first screen of the questionnaire. [12] Number consecutively and organize questions according on content and structure. [14] Organize the questionnaire in order to facilitate questionnaire flow. [9, 16] Ask one question at time. [9] Use an item-in-a-series format to place items with the same response categories. [16] Include useful graphical languages to facilitate user’s navigation through the desired response path. [16] Present and organize the questionnaire so that it is attractive and easy to complete, without including too much information for page and align elements horizontally or vertically in order to facilitate the reading. [16] Include delicate, tricky and the most important questions at 1/3 of the questionnaire, when the user has already start to complete it, but he’s not yet bored. [12] Include open-ended questions at the 2/3 of the questionnaire, in order to maintain the users’ interest. [12] If the topic is the same, include open-ended questions before closed-ended questions, to avoid influencing respondents with the fixed option choices. [12] Do not separate a question from its response set. [12] Avoid to require that a user provide an answer to one question before moving on to the next question. [12] In order to reduce survey bias, directs users’ navigation, requiring them to answer questions in the order intended by the study designer. [12] The initial question must be fully visible on the first screen of the questionnaire, interest-getting, easily comprehended and answered by all respondents. Do not use drop-down box or require scrolling in order to see the entire first question. [12]
	*Layout*	Pay attention to spatial arrangement, color, brightness and consistency, when you create the visual presentation of a questionnaire. [20] It is better to create a plain questionnaire than a fancy version of the same questionnaire, because a plain version increases response rate, the completeness and reduces completion time. [9] Limit the use of matrices where necessary. [5] In order to ensure an easy reading use a consistent figure/ground format and limit the use of reverse print to section headings and/or question numbers. [16] Try to respect the consistency of the questionnaire also in the direction in which the scales are displayed. [16] It is better to use shorter lines to avoid words’ skip. [16] Highlight, but sparingly, words and phrases that indicate item’s change. [16] Make sure that questionnaire’s visual appearance is consistent to the designer’s measurement intentions and to the aim of the survey. [16] Use simple construction techniques, in order to not require multiple steps to answer items. [16] Design the questionnaire using a conventional formula similar to those normally used on paper self administered questionnaires. [16] Use the most challenging system, when administering the questionnaire through a PDA used as personal organizer. [16] Write sentences with no more than 20 words and with no more than 75 characters per line. Include paragraphs with no more than 5 sentences. [11] Do not divide the questionnaire in too many sections, in order to not confuse the users and make always clear survey’s focus. [12] Design simple and not overcrowded pages. Do not use pop-up windows, rollover text, cascading menus, and new browser windows; if pop-up windows are used, make sure that the default option is the most forgiving and if new browser windows are opened, make sure that a simple means to get back to the original is provided. [12] Choose a layout that don’t influence users’ opinions and answers. [12] Do not choose a columnar format with two-column design or multiple columns to present questionnaire’s text, in order to prevent that the user choose the wrong questions’ grid, and to avoid that the users forget to respond to subsequent columns before moving to the next page. [14] Design the questionnaire based on the question types supported (single choice, multi-choice, open ends). [18]	Pay attention to spatial arrangement, color, brightness and consistency, when you create the visual presentation of a questionnaire. [10] It is better to create a plain questionnaire than a fancy version of the same questionnaire, because a plain version increases response rate, the completeness and reduces completion time. [9] Limit the use of matrices where necessary. [5] In order to ensure an easy reading use a consistent figure/ground format and limit the use of reverse print to section headings and/or question numbers. [16] Try to respect the consistency of the questionnaire also in the direction in which the scales are displayed. [16] It is better to use shorter lines to avoid words’ skip. [16] Highlight, but sparingly, words and phrases that indicate item’s change. [16] Make sure that questionnaire’s visual appearance is consistent to the designer’s measurement intentions and to the aim of the survey. [16] Use simple construction techniques, in order to not require multiple steps to answer items. [16] Design the questionnaire using a conventional formula similar to those normally used on paper self administered questionnaires. [16] Use the most challenging system, when administering the questionnaire through a PDA used as personal organizer. [16] Write sentences with no more than 20 words and with no more than 75 characters per line. Include paragraphs with no more than 5 sentences. [11] Do not divide the questionnaire in too many sections, in order to not confuse the users and make always clear survey’s focus. [12] Design simple and not overcrowded pages. Do not use pop-up windows, rollover text, cascading menus, and new browser windows; if pop-up windows are used, make sure that the default option is the most forgiving and if new browser windows are opened, make sure that a simple means to get back to the original is provided. [12] Choose a layout that don’t influence users’ opinions and answers. [12] Do not choose a columnar format with two-column design or multiple columns to present questionnaire’s text, in order to prevent that the user choose the wrong questions’ grid, and to avoid that the users forget to respond to subsequent columns before moving to the next page. [14] Design the questionnaire based on the question types supported (single choice, multi-choice, open ends). [18]	Pay attention to spatial arrangement, color, brightness and consistency, when you create the visual presentation of a questionnaire. [10] It is better to create a plain questionnaire than a fancy version of the same questionnaire, because a plain version increases response rate, the completeness and reduces completion time. [9] Limit the use of matrices where necessary. [5] In order to ensure an easy reading use a consistent figure/ground format and limit the use of reverse print to section headings and/or question numbers. [16] Try to respect the consistency of the questionnaire also in the direction in which the scales are displayed. [16] It is better to use shorter lines to avoid words’ skip. [16] Highlight, but sparingly, words and phrases that indicate item’s change. [16] Make sure that questionnaire’s visual appearance is consistent to the designer’s measurement intentions and to the aim of the survey. [16] Use simple construction techniques, in order to not require multiple steps to answer items. [16] Design the questionnaire using a conventional formula similar to those normally used on paper self administered questionnaires. [16] Use the most challenging system, when administering the questionnaire through a PDA used as personal organizer. [16] Write sentences with no more than 20 words and with no more than 75 characters per line. Include paragraphs with no more than 5 sentences. [11] Do not divide the questionnaire in too many sections, in order to not confuse the users and make always clear survey’s focus. [12] Design simple and not overcrowded pages. Do not use pop-up windows, rollover text, cascading menus, and new browser windows; if pop-up windows are used, make sure that the default option is the most forgiving and if new browser windows are opened, make sure that a simple means to get back to the original is provided. [12] Choose a layout that don’t influence users’ opinions and answers. [12] Do not choose a columnar format with two-column design or multiple columns to present questionnaire’s text, in order to prevent that the user choose the wrong questions’ grid, and to avoid that the users forget to respond to subsequent columns before moving to the next page. [14] Design the questionnaire based on the question types supported (single choice, multi-choice, open ends). [18]	Pay attention to spatial arrangement, color, brightness and consistency, when you create the visual presentation of a questionnaire. [10] It is better to create a plain questionnaire than a fancy version of the same questionnaire, because a plain version increases response rate, the completeness and reduces completion time. [9] Limit the use of matrices where necessary. [5] In order to ensure an easy reading use a consistent figure/ground format and limit the use of reverse print to section headings and/or question numbers. [16] Try to respect the consistency of the questionnaire also in the direction in which the scales are displayed. [16] It is better to use shorter lines to avoid words’ skip. [16] Highlight, but sparingly, words and phrases that indicate item’s change. [16] Make sure that questionnaire’s visual appearance is consistent to the designer’s measurement intentions and to the aim of the survey. [16] Use simple construction techniques, in order to not require multiple steps to answer items. [16] Design the questionnaire using a conventional formula similar to those normally used on paper self administered questionnaires. [16] Use the most challenging system, when administering the questionnaire through a PDA used as personal organizer. [16] Write sentences with no more than 20 words and with no more than 75 characters per line. Include paragraphs with no more than 5 sentences. [11] Do not divide the questionnaire in too many sections, in order to not confuse the users and make always clear survey’s focus. [12] Design simple and not overcrowded pages. Do not use pop-up windows, rollover text, cascading menus, and new browser windows; if pop-up windows are used, make sure that the default option is the most forgiving and if new browser windows are opened, make sure that a simple means to get back to the original is provided. [12] Choose a layout that don’t influence users’ opinions and answers. [12] In mobile questionnaire, it is better to fit the question on the screen including the entire scale and all its items. If this is not possible, check that the respondent can effectively scroll the question or zoom in and out to gain an overview of all options. [14] Consider the number of points in the scale carefully: 5-point scales is convenient for a mobile phone screen (size 3,5”), since they fitted the screen easily with a readable font size. [17] Make selecting an icon or item for answering easy. On touch screen devices, use large enough icons and adequate spacing between items, so that the neighboring items or icons are not selected by accident. [17] Do not choose a columnar format with two-column design or multiple columns to present questionnaire’s text, in order to prevent that the user choose the wrong questions’ grid, and to avoid that the users forget to respond to subsequent columns before moving to the next page. [17] Design the questionnaire based on the question types supported (single choice, multi-choice, open ends). [18]
	*Scrolling*	Chose scroll, page by page, or mixed construction on the base of measurement and response considerations. [10] Do not include question structures that require scrolling or toggling between screens. [16] Do not use scroll bars in ePRO applications, since it is better to make all information simultaneously available on the screen. [16] Limit the need for scrolling, in particular design the welcome page into a single screen so it is not necessary to scroll. [11] Inform the users of the need to scroll. [12] Use jump buttons as alternative to scroll bars, to guide the users to the next screen. [12] Prefer the use of paging function like “page up” and “page down” keys as alternative to scrolling. [12]	Chose scroll, page by page, or mixed construction on the base of measurement and response considerations. [2] Do not include question structures that require scrolling or toggling between screens. [16] Do not use scroll bars in ePRO applications, since it is better to make all information simultaneously available on the screen. [16] Limit the need for scrolling, in particular design the welcome page into a single screen so it is not necessary to scroll. [11] Inform the users of the need to scroll. [12] Use jump buttons as alternative to scroll bars, to guide the users to the next screen. [12] Prefer the use of paging function like “page up” and “page down” keys as alternative to scrolling. [12] Design web questionnaires so they scroll from question to question without order mistakes. [2] Reduce the need of scrolling, and think to use the hyperlink to facilitate the change from one question to another one. [14]	Chose scroll, page by page, or mixed construction on the base of measurement and response considerations. [14] Do not include question structures that require scrolling or toggling between screens. [16] Do not use scroll bars in ePRO applications, since it is better to make all information simultaneously available on the screen. [16] Limit the need for scrolling, in particular design the welcome page into a single screen so it is not necessary to scroll. [11] Inform the users of the need to scroll. [12] Use jump buttons as alternative to scroll bars, to guide the users to the next screen. [12] Prefer the use of paging function like “page up” and “page down” keys as alternative to scrolling. [12] Design web questionnaires so they scroll from question to question without order mistakes. [2] Reduce the need of scrolling, and think to use the hyperlink to facilitate the change from one question to another one. [14]	Chose scroll, page by page, or mixed construction on the base of measurement and response considerations. [14] Do not include question structures that require scrolling or toggling between screens. [16] Limit the need for scrolling, in particular design the welcome page into a single screen so it is not necessary to scroll. [16] Inform the users of the need to scroll. [12] Use jump buttons as alternative to scroll bars, to guide the users to the next screen. [12] Prefer the use of paging function like “page up” and “page down” keys as alternative to scrolling. [12] Design web questionnaires so they scroll from question to question without order mistakes. [2] Reduce the need of scrolling, and think to use the hyperlink to facilitate the change from one question to another one. [2]
	*Font size and style*	Use a font style and size easy to read, like Arial 10–12 point. [14] Use bold type, shading and broad lines to help direct respondents’ attention. [9] Use a font size of 12 point. [9] Use readable and familiar fonts, presenting mixed case text or standard sentence formatting. [11] Use upper case or all capitals only for emphasis. [12] Test font colors and size with a screen magnifier before questionnaire distribution. [12]	Use a font style and size easy to read, like Arial 10–12 point. [12] Use bold type, shading and broad lines to help direct respondents’ attention. [9] Use a font size of 12 point. [9] Use readable and familiar fonts, presenting mixed case text or standard sentence formatting. [11] Use upper case or all capitals only for emphasis. [12] Test font colors and size with a screen magnifier before questionnaire distribution. [12]	Use a font style and size easy to read, like Arial 10–12 point. [12] Use bold type, shading and broad lines to help direct respondents’ attention. [9] Use a font size of 12 point. [9] Use readable and familiar fonts, presenting mixed case text or standard sentence formatting. [11] Use upper case or all capitals only for emphasis. [12] Test font colors and size with a screen magnifier before questionnaire distribution. [12]	Use a font style and size easy to read, like Arial 10–12 point. [12] Use bold type, shading and broad lines to help direct respondents’ attention. [9] Use a font size of 12 point. [9] Use readable and familiar fonts, presenting mixed case text or standard sentence formatting. [11] Use upper case or all capitals only for emphasis. [12] Test font colors and size with a screen magnifier before questionnaire distribution. [12] Ensure that is not necessary to zooming to read the text: it may be difficult due to the device used or due to context related issues. [12]
	*Color*	It is better to use dark print for questions and light print for answer choices. [17] Use a lightly shaded background colors on which to write all questions, and evidence all answer spaces in white in order to reduce item non-response. [16] Do not use red and green colors for necessary information, but only to enhance or clarify screen display. [16] Use consistent color coding throughout the questionnaire. [11] Use a neutral background color in order to make text very easy to read. [12] Use colors of high contrast when pairing colors or using two colors in close proximity. [12] Consider standard color associations among different cultures. [12] Limit the use of color in order to maintain figure/ground consistency and read-ability and consent an easy navigational flow. [12]	It is better to use dark print for questions and light print for answer choices. [14] Use a lightly shaded background colors on which to write all questions, and evidence all answer spaces in white in order to reduce item non-response. [16] Do not use red and green colors for necessary information, but only to enhance or clarify screen display. [16] Use consistent color coding throughout the questionnaire. [11] Use a neutral background color in order to make text very easy to read. [12] Use colors of high contrast when pairing colors or using two colors in close proximity. [12] Consider standard color associations among different cultures. [12] Limit the use of color in order to maintain figure/ground consistency and read-ability and consent an easy navigational flow. [12]	It is better to use dark print for questions and light print for answer choices. [14] Use a lightly shaded background colors on which to write all questions, and evidence all answer spaces in white in order to reduce item non-response. [16] Do not use red and green colors for necessary information, but only to enhance or clarify screen display. [16] Use consistent color coding throughout the questionnaire. [11] Use a neutral background color in order to make text very easy to read. [12] Use colors of high contrast when pairing colors or using two colors in close proximity. [12] Consider standard color associations among different cultures. [12] Limit the use of color in order to maintain figure/ground consistency and read-ability and consent an easy navigational flow. [12]	It is better to use dark print for questions and light print for answer choices. [14] Use a lightly shaded background colors on which to write all questions, and evidence all answer spaces in white in order to reduce item non-response. [16] Do not use red and green colors for necessary information, but only to enhance or clarify screen display. [16] Use consistent color coding throughout the questionnaire. [11] Use a neutral background color in order to make text very easy to read. [12] Use colors of high contrast when pairing colors or using two colors in close proximity. [12] Consider standard color associations among different cultures. [12] Limit the use of color in order to maintain figure/ground consistency and read-ability and consent an easy navigational flow. [12]
	*Questions’ graphic*	Dispose questions in the upper left quadrant. Information not needed by respondent must be placed into the lower left quadrant. [14] Evidence the beginning of each question in a consistent way. [16] Include blank space between the questions; less blank space should be placed between subcomponents of the same question. [16] Aligns vertically the question’s subcomponents in order to make easier the response task. [16] Check that there is no difference in the visual appearance of questions resulting from different screen configurations, operating systems, browsers, partial screen displays, and wrap-around text. [16] Make sure that questions’ format is distinguishable from instructions’ and answers’ format. [16] Separate clearly the question stem from the answer spaces. [13] Present questions in a conventional format similar to paper questionnaires’ format. [20] Layout questions as they are easy to read for the users, and evidence the most important keywords. [20] Evidence the references between question and corresponding answer. [14] Make clearly the difference between questions and answers with similarly format. [14]	Dispose questions in the upper left quadrant. Information not needed by respondent must be placed into the lower left quadrant. [14] Evidence the beginning of each question in a consistent way. [16] Include blank space between the questions; less blank space should be placed between subcomponents of the same question. [16] Aligns vertically the question’s subcomponents in order to make easier the response task. [16] Check that there is no difference in the visual appearance of questions resulting from different screen configurations, operating systems, browsers, partial screen displays, and wrap-around text. [16] Make sure that questions’ format is distinguishable from instructions’ and answers’ format. [16] Separate clearly the question stem from the answer spaces. [13] Present questions in a conventional format similar to paper questionnaires’ format. [20] Layout questions as they are easy to read for the users, and evidence the most important keywords. [20] Evidence the references between question and corresponding answer. [14] Make clearly the difference between questions and answers with similarly format. [14]	Dispose questions in the upper left quadrant. Information not needed by respondent must be placed into the lower left quadrant. [14] Evidence the beginning of each question in a consistent way. [16] Include blank space between the questions; less blank space should be placed between subcomponents of the same question. [16] Aligns vertically the question’s subcomponents in order to make easier the response task. [16] Check that there is no difference in the visual appearance of questions resulting from different screen configurations, operating systems, browsers, partial screen displays, and wrap-around text. [16] Make sure that questions’ format is distinguishable from instructions’ and answers’ format. [16] Separate clearly the question stem from the answer spaces. [13] Present questions in a conventional format similar to paper questionnaires’ format. [20] Layout questions as they are easy to read for the users, and evidence the most important keywords. [20] Evidence the references between question and corresponding answer. [14] Make clearly the difference between questions and answers with similarly format. [14]	Dispose questions in the upper left quadrant. Information not needed by respondent must be placed into the lower left quadrant. [14] Evidence the beginning of each question in a consistent way. [16] Include blank space between the questions; less blank space should be placed between subcomponents of the same question. [16] Aligns vertically the question’s subcomponents in order to make easier the response task. [16] Check that there is no difference in the visual appearance of questions resulting from different screen configurations, operating systems, browsers, partial screen displays, and wrap-around text. [16] Make sure that questions’ format is distinguishable from instructions’ and answers’ format. [16] Separate clearly the question stem from the answer spaces. [13] Present questions in a conventional format similar to paper questionnaires’ format. [20] Layout questions as they are easy to read for the users, and evidence the most important keywords. [20] Evidence the references between question and corresponding answer. [14] Make clearly the difference between questions and answers with similarly format. [14]
	*Answers’ graphic*	Receive the response options on separate lines. [14] Place answer categories vertically. [9] Place answer spaces consistently to the left or right of the category labels. [16] To facilitate the answer process, use numbers or simple answer boxes. [16] Ensure that space between answer choices is consistent with measurement intent. [16] Ensure that the area to be tapped is large enough to make easy the selection of a response option. [16] Make immediately visible the users’ answer to questions in order to evidence the effect of their actions. [11] Design answer items so are clearly related to the proper questions. [12]	Receive the response options on separate lines. [14] Place answer categories vertically. [9] Place answer spaces consistently to the left or right of the category labels. [16] To facilitate the answer process, use numbers or simple answer boxes. [16] Ensure that space between answer choices is consistent with measurement intent. [16] Ensure that the area to be tapped is large enough to make easy the selection of a response option. [16] Make immediately visible the users’ answer to questions in order to evidence the effect of their actions. [11] Design answer items so are clearly related to the proper questions. [12]	Receive the response options on separate lines. [14] Place answer categories vertically. [9] Place answer spaces consistently to the left or right of the category labels. [16] To facilitate the answer process, use numbers or simple answer boxes. [16] Ensure that space between answer choices is consistent with measurement intent. [16] Ensure that the area to be tapped is large enough to make easy the selection of a response option. [16] Make immediately visible the users’ answer to questions in order to evidence the effect of their actions. [11] Design answer items so are clearly related to the proper questions. [12]	Receive the response options on separate lines. [14] Place answer categories vertically. [9] Place answer spaces consistently to the left or right of the category labels. [16] To facilitate the answer process, use numbers or simple answer boxes. [16] Ensure that space between answer choices is consistent with measurement intent. [16] Ensure that the area to be tapped is large enough to make easy the selection of a response option. [16] Make immediately visible the users’ answer to questions in order to evidence the effect of their actions. [11] Design answer items so are clearly related to the proper questions. [12]
	*Symbols and imagines*	It is better to use large and bright symbols to identify the starting point on each page. [14] Use graphical languages to facilitate the logical flow of the questionnaire. [16] Consider the different meanings of symbols among different cultures. [16] Use the graphic only to increase the user’s motivation, download speed improvement and tiredness reduction. [11] Ensure that pictures and graphics not lead to misunderstanding. [6] Use hyper text, colors, interaction strategies and other Web communication capabilities to create more attraction. [6] Keep the number of graphics at minimum to avoid increasing download time and making difficult the accessibility of questionnaires across the sample. [6] Prefer small graphics that will download quickly: individual images should not exceed 5KB in size and single web-page should not exceed 20KB of graphics in total. [12] Include a text only version of the webpage, in addition that with graphics. [12] Use crisp and clear images to benefit users with visual disabilities. Remember to not blur pictures, grey out information or overlap menus. [12] Make sure that users can skip multimedia content without penalty. It is better to avoid multimedia and audio clips with graphics if users have to download the plug ins to complete the questionnaire. [12] Use logotypes in order to reduce item non-response and the tendency of users to choose the middle point of a rating scale. [12] The use of graphical symbols or words is useful to indicate user’s progress through questionnaire. [2] Avoid graphics and symbols that require significant increase in computer memory. [14]	It is better to use large and bright symbols to identify the starting point on each page. [14] Use graphical languages to facilitate the logical flow of the questionnaire. [16] Consider the different meanings of symbols among different cultures. [16] Use the graphic only to increase the user’s motivation, download speed improvement and tiredness reduction. [11] Ensure that pictures and graphics not lead to misunderstanding. [6] Use hyper text, colors, interaction strategies and other Web communication capabilities to create more attraction. [6] Keep the number of graphics at minimum to avoid increasing download time and making difficult the accessibility of questionnaires across the sample. [6] Prefer small graphics that will download quickly: individual images should not exceed 5KB in size and single web-page should not exceed 20KB of graphics in total. [12] Include a text only version of the webpage, in addition that with graphics. [12] Use crisp and clear images to benefit users with visual disabilities. Remember to not blur pictures, grey out information or overlap menus. [12] Make sure that users can skip multimedia content without penalty. It is better to avoid multimedia and audio clips with graphics if users have to download the plug ins to complete the questionnaire. [12] Use logotypes in order to reduce item non-response and the tendency of users to choose the middle point of a rating scale. [12] The use of graphical symbols or words is useful to indicate user’s progress through questionnaire. [2] Avoid graphics and symbols that require significant increase in computer memory. [14]	It is better to use large and bright symbols to identify the starting point on each page. [14] Use graphical languages to facilitate the logical flow of the questionnaire. [16] Consider the different meanings of symbols among different cultures. [16] Use the graphic only to increase the user’s motivation, download speed improvement and tiredness reduction. [11] Ensure that pictures and graphics not lead to misunderstanding. [6] Use hyper text, colors, interaction strategies and other Web communication capabilities to create more attraction. [6] Keep the number of graphics at minimum to avoid increasing download time and making difficult the accessibility of questionnaires across the sample. [6] Prefer small graphics that will download quickly: individual images should not exceed 5KB in size and single web-page should not exceed 20KB of graphics in total. [12] Include a text only version of the webpage, in addition that with graphics. [12] Use crisp and clear images to benefit users with visual disabilities. Remember to not blur pictures, grey out information or overlap menus. [12] Make sure that users can skip multimedia content without penalty. It is better to avoid multimedia and audio clips with graphics if users have to download the plug ins to complete the questionnaire. [12] Use logotypes in order to reduce item non-response and the tendency of users to choose the middle point of a rating scale. [12] The use of graphical symbols or words is useful to indicate user’s progress through questionnaire. [2] Avoid graphics and symbols that require significant increase in computer memory. [14]	It is better to use large and bright symbols to identify the starting point on each page. [14] Use graphical languages to facilitate the logical flow of the questionnaire. [16] Consider the different meanings of symbols among different cultures. [16] Use the graphic only to increase the user’s motivation, download speed improvement and tiredness reduction. [11] Ensure that pictures and graphics not lead to misunderstanding. [6] Use hyper text, colors, interaction strategies and other Web communication capabilities to create more attraction. [6] Keep the number of graphics at minimum to avoid increasing download time and making difficult the accessibility of questionnaires across the sample. [6] Prefer small graphics that will download quickly: individual images should not exceed 5KB in size and single web-page should not exceed 20KB of graphics in total. [12] Include a text only version of the webpage, in addition that with graphics. [12] Use crisp and clear images to benefit users with visual disabilities. Remember to not blur pictures, grey out information or overlap menus. [12] Make sure that users can skip multimedia content without penalty. It is better to avoid multimedia and audio clips with graphics if users have to download the plug ins to complete the questionnaire. [12] Use logotypes in order to reduce item non-response and the tendency of users to choose the middle point of a rating scale. [12] The use of graphical symbols or words is useful to indicate user’s progress through questionnaire. [2] Avoid graphics and symbols that require significant increase in computer memory. [14]
	*Tables and frame*	Use tables to present ordinal responses of several constructs within a single question. [14] Do not use frames, since they can make pages difficult to read and print, increase load time, and cause problems for respondents who use assistive technology. [9] Avoid frame technology, because it distracts the respondents from answering the questions. [12]	Use tables to present ordinal responses of several constructs within a single question. [14] Do not use frames, since they can make pages difficult to read and print, increase load time, and cause problems for respondents who use assistive technology. [9] Avoid frame technology, because it distracts the respondents from answering the questions. [12]	Use tables to present ordinal responses of several constructs within a single question. [14] Do not use frames, since they can make pages difficult to read and print, increase load time, and cause problems for respondents who use assistive technology. [9] Avoid frame technology, because it distracts the respondents from answering the questions. [12]	Use tables to present ordinal responses of several constructs within a single question. [14] Do not use frames, since they can make pages difficult to read and print, increase load time, and cause problems for respondents who use assistive technology. [9] Avoid frame technology, because it distracts the respondents from answering the questions. [12]
	*Visual analogue scale*	Do not include initial starting position for the cursor, in order to reduce the risk of bias within the subjective assessment. [14] The line should start blank and a mark appear where the scale is first tapped. Then the mark may be dragged with the cursor to adjust it, or the position of a second tap may replace the first, or both. [11]	Do not include initial starting position for the cursor, in order to reduce the risk of bias within the subjective assessment. [11] The line should start blank and a mark appear where the scale is first tapped. Then the mark may be dragged with the cursor to adjust it, or the position of a second tap may replace the first, or both. [11]	Do not include initial starting position for the cursor, in order to reduce the risk of bias within the subjective assessment. [11] The line should start blank and a mark appear where the scale is first tapped. Then the mark may be dragged with the cursor to adjust it, or the position of a second tap may replace the first, or both. [11]	Do not include initial starting position for the cursor, in order to reduce the risk of bias within the subjective assessment. [11] The line should start blank and a mark appear where the scale is first tapped. Then the mark may be dragged with the cursor to adjust it, or the position of a second tap may replace the first, or both. [11]
	*Matrix questions*	Matrix questions must be used sparingly to condense and simplify a question if it involves many response options. [11] Do not use matrix questions in tabular form in order to avoid that respondents might possibly choose those answers that are easiest to reach with the mouse, rather than those buttons that express their real opinion. [12]	Matrix questions must be used sparingly to condense and simplify a question if it involves many response options. [14] Do not use matrix questions in tabular form in order to avoid that respondents might possibly choose those answers that are easiest to reach with the mouse, rather than those buttons that express their real opinion. [12]	Matrix questions must be used sparingly to condense and simplify a question if it involves many response options. [14] Do not use matrix questions in tabular form in order to avoid that respondents might possibly choose those answers that are easiest to reach with the mouse, rather than those buttons that express their real opinion. [12]	Matrix questions must be used sparingly to condense and simplify a question if it involves many response options. [14] Do not use matrix questions in tabular form in order to avoid that respondents might possibly choose those answers that are easiest to reach with the mouse, rather than those buttons that express their real opinion. [12]
	*Drop down boxes*	Drop down boxes should be used only when answering process is simplified and should be identified with click here. [14] Avoid drop-down selections, because the options are not available when the selection is being reviewed by the patient. [16] Use drop-down boxes sparingly, only when very long lists of response options are required. [11] Enhance drop-down boxes’ usability by incorporating type-ahead look up and avoiding requirement for, or possibility of, multiple selections. [12] It is important that first option in the drop-down box is not visible by default since this can lead respondents and that visual clues to indicate how the drop-box is used are clear and visible. [12]	Drop down boxes should be used only when answering process is simplified and should be identified with click here. [12] Avoid drop-down selections, because the options are not available when the selection is being reviewed by the patient. [16] Use drop-down boxes sparingly, only when very long lists of response options are required. [11] Enhance drop-down boxes’ usability by incorporating type-ahead look up and avoiding requirement for, or possibility of, multiple selections. [12] It is important that first option in the drop-down box is not visible by default since this can lead respondents and that visual clues to indicate how the drop-box is used are clear and visible. [12]	Drop down boxes should be used only when answering process is simplified and should be identified with click here. [12] Avoid drop-down selections, because the options are not available when the selection is being reviewed by the patient. [16] Use drop-down boxes sparingly, only when very long lists of response options are required. [11] Enhance drop-down boxes’ usability by incorporating type-ahead look up and avoiding requirement for, or possibility of, multiple selections. [12] It is important that first option in the drop-down box is not visible by default since this can lead respondents and that visual clues to indicate how the drop-box is used are clear and visible. [12]	Drop down boxes should be used only when answering process is simplified and should be identified with click here. [12] Avoid drop-down selections, because the options are not available when the selection is being reviewed by the patient. [16] Use drop-down boxes sparingly, only when very long lists of response options are required. [11] Enhance drop-down boxes’ usability by incorporating type-ahead look up and avoiding requirement for, or possibility of, multiple selections. [12] It is important that first option in the drop-down box is not visible by default since this can lead respondents and that visual clues to indicate how the drop-box is used are clear and visible. [12]
	*Radio buttons*	In compares to entry boxes, the use of radio buttons may decrease the likelihood of missing data. [12] Radio buttons reduce missing items, whereas text box entries increase the quality of responses. [9] The area available to be tapped must be explicitly indicated, as with the lower option. [5] When the radio button is selected, the whole button must be highlighted, and the text must remain visible. [11] Only one radio button within any given group of radio buttons can be selected at a time. [11] Provide clear instructions when using radio buttons, especially for users with limited computer experience, who often do not know how to provide and/or change their responses. [12]	In compares to entry boxes, the use of radio buttons may decrease the likelihood of missing data. [12] Radio buttons reduce missing items, whereas text box entries increase the quality of responses. [9] The area available to be tapped must be explicitly indicated, as with the lower option. [5] When the radio button is selected, the whole button must be highlighted, and the text must remain visible. [11] Only one radio button within any given group of radio buttons can be selected at a time. [11] Provide clear instructions when using radio buttons, especially for users with limited computer experience, who often do not know how to provide and/or change their responses. [12]	In compares to entry boxes, the use of radio buttons may decrease the likelihood of missing data. [12] Radio buttons reduce missing items, whereas text box entries increase the quality of responses. [9] The area available to be tapped must be explicitly indicated, as with the lower option. [5] When the radio button is selected, the whole button must be highlighted, and the text must remain visible. [11] Only one radio button within any given group of radio buttons can be selected at a time. [11] Provide clear instructions when using radio buttons, especially for users with limited computer experience, who often do not know how to provide and/or change their responses. [12]	In compares to entry boxes, the use of radio buttons may decrease the likelihood of missing data. [12] Radio buttons reduce missing items, whereas text box entries increase the quality of responses. [9] The area available to be tapped must be explicitly indicated, as with the lower option. [5] When the radio button is selected, the whole button must be highlighted, and the text must remain visible. [11] Only one radio button within any given group of radio buttons can be selected at a time. [11] Provide clear instructions when using radio buttons, especially for users with limited computer experience, who often do not know how to provide and/or change their responses. [12]
	*Check buttons*	Use short entry boxes, rather than long entry boxes in order to reduce the likelihood of invalid responses. [12] In case of multiple selections present the alternatives as boxes to be tapped, in order to make the area larger, and to explicit the “none of the above” option. [9] Spin button must have a large area to be tapped, it should not start at zero, but with a blank or null value. [11] Allow many selections within a set when multiple response options can be selected. [11] Clear instructions should be provided when using check boxes, in order to consent the participation of all users, including users with disabilities. [12]	Use short entry boxes, rather than long entry boxes in order to reduce the likelihood of invalid responses. [12] In case of multiple selections present the alternatives as boxes to be tapped, in order to make the area larger, and to explicit the “none of the above” option. [9] Spin button must have a large area to be tapped, it should not start at zero, but with a blank or null value. [11] Allow many selections within a set when multiple response options can be selected. [11] Clear instructions should be provided when using check boxes, in order to consent the participation of all users, including users with disabilities. [12]	Use short entry boxes, rather than long entry boxes in order to reduce the likelihood of invalid responses. [12] In case of multiple selections present the alternatives as boxes to be tapped, in order to make the area larger, and to explicit the “none of the above” option. [9] Spin button must have a large area to be tapped, it should not start at zero, but with a blank or null value. [11] Allow many selections within a set when multiple response options can be selected. [11] Clear instructions should be provided when using check boxes, in order to consent the participation of all users, including users with disabilities. [12]	Use short entry boxes, rather than long entry boxes in order to reduce the likelihood of invalid responses. [12] In case of multiple selections present the alternatives as boxes to be tapped, in order to make the area larger, and to explicit the “none of the above” option. [9] Spin button must have a large area to be tapped, it should not start at zero, but with a blank or null value. [11] Allow many selections within a set when multiple response options can be selected. [11] Clear instructions should be provided when using check boxes, in order to consent the participation of all users, including users with disabilities. [12]
**INFORMATIC ACCESSIBILITY**	*Questionnaire distribution and login procedures*	Use a mixed-mode strategy, (electronic and pen-and-paper questionnaire), to reach respondents without access to the Internet. [12] Inform and encourage the sample to answer, through sending an E-mail to each person or even to a certain group, putting questionnaire link on the web page, using Popup window and brochure distribution. Even sending reminder letters with an appropriate scheduling should be considered. [5] Use a semiautomatic login procedure, including an access code, in order to not decrease response rates and increase data quality. [6] Develop a identification system to control coverage error and highlight the reliability of the test. [5] Restrict the accesses to the questionnaire, using an ID and a password. [16] If the questionnaire is directed to a specific sample, include a registration or login screen. [6] Give a PIN number only to the sample, in order to avoid questionnaire access by other people. [12]	Use a mixed-mode strategy, (electronic and pen-and-paper questionnaire), to reach respondents without access to the Internet. [14] Inform and encourage the sample to answer, through sending an E-mail to each person or even to a certain group, putting questionnaire link on the web page, using Popup window and brochure distribution. Even sending reminder letters with an appropriate scheduling should be considered. [5] Use a semiautomatic login procedure, including an access code, in order to not decrease response rates and increase data quality. [6] The distribution of web survey to anyone willing to complete them can be considered a form of indirect mailing. Such surveys are prone to various form of bias. [5] Develop a identification system to control coverage error and highlight the reliability of the test. [5] Restrict the accesses to the questionnaire, using an ID and a password. [16] If the questionnaire is directed to a specific sample, include a registration or login screen. [6] Give a PIN number only to the sample, in order to avoid questionnaire access by other people. [12]	Use a mixed-mode strategy, (electronic and pen-and-paper questionnaire), to reach respondents without access to the Internet. [14] Inform and encourage the sample to answer, through sending an E-mail to each person or even to a certain group, putting questionnaire link on the web page, using Popup window and brochure distribution. Even sending reminder letters with an appropriate scheduling should be considered. [5] Use a semiautomatic login procedure, including an access code, in order to not decrease response rates and increase data quality. [6] The distribution of web survey to anyone willing to complete them can be considered a form of indirect mailing. Such surveys are prone to various form of bias. [5] Develop a identification system to control coverage error and highlight the reliability of the test. [5] Restrict the accesses to the questionnaire, using an ID and a password. [16] If the questionnaire is directed to a specific sample, include a registration or login screen. [6] Give a PIN number only to the sample, in order to avoid questionnaire access by other people. [12]	Use a mixed-mode strategy, (electronic and pen-and-paper questionnaire), to reach respondents without access to the Internet. [14] Inform and encourage the sample to answer, through sending an E-mail to each person or even to a certain group, putting questionnaire link on the web page, using Popup window and brochure distribution. Even sending reminder letters with an appropriate scheduling should be considered. [5] Manage deployment process when email address for mobile surveys is different from main panel email address. [6] Use a semiautomatic login procedure, including an access code, in order to not decrease response rates and increase data quality. [10] The distribution of web survey to anyone willing to complete them can be considered a form of indirect mailing. Such surveys are prone to various form of bias. [5] Develop a identification system to control coverage error and highlight the reliability of the test. [5] Restrict the accesses to the questionnaire, using an ID and a password. [16] If the questionnaire is directed to a specific sample, include a registration or login screen. [6] Give a PIN number only to the sample, in order to avoid questionnaire access by other people. [12]
	*Cover letter*	Cover letter should state the objective of the survey and highlight why potential respondents were selected. [14] Provide an estimate of the time required to complete the questionnaire. [9] Use a personalize cover letter to increases survey response rate. [9]	Cover letter should state the objective of the survey and highlight why potential respondents were selected. [5, 9] Provide an estimate of the time required to complete the questionnaire. [9] Potential respondents can be sent an electronic cover letter either with the initial or reminder questionnaires attached or with a link to the Internet-based questionnaire. Alternatively, the cover letter and questionnaire can be posted on the Web. [9] Use a personalize cover letter to increases survey response rate. [9]	Cover letter should state the objective of the survey and highlight why potential respondents were selected. [5, 9] Provide an estimate of the time required to complete the questionnaire. [9] Potential respondents can be sent an electronic cover letter either with the initial or reminder questionnaires attached or with a link to the Internet-based questionnaire. Alternatively, the cover letter and questionnaire can be posted on the Web. [9] Use a personalize cover letter to increases survey response rate. [9]	Cover letter should state the objective of the survey and highlight why potential respondents were selected. [5, 9] Provide an estimate of the time required to complete the questionnaire. [9] Use a personalize cover letter to increases survey response rate. [9]
	*Thank you page and user’s feedback*	Take in consideration the email correspondence from respondents, because it can be an important source of information. [5, 9] Remember to read and answer to feedback messages sanded by the patients. [5] At the end of the questionnaire, remember to thank the users for their collaboration, using a gentle and friendly tone. [11]	Take in consideration the email correspondence from respondents, because it can be an important source of information. [12] Remember to read and answer to feedback messages sanded by the patients. [5] At the end of the questionnaire, remember to thank the users for their collaboration, using a gentle and friendly tone. [11]	Take in consideration the email correspondence from respondents, because it can be an important source of information. [12] Remember to read and answer to feedback messages sanded by the patients. [5] At the end of the questionnaire, remember to thank the users for their collaboration, using a gentle and friendly tone. [11]	Take in consideration the email correspondence from respondents, because it can be an important source of information. [12] Remember to read and answer to feedback messages sanded by the patients. [5] At the end of the questionnaire, remember to thank the users for their collaboration, using a gentle and friendly tone. [11]
	*Confirmation message*	Include confirmation message that an action has been successfully completed where there is a significant possibility that it will not do so. [12]	Include confirmation message that an action has been successfully completed where there is a significant possibility that it will not do so. [11]	Include confirmation message that an action has been successfully completed where there is a significant possibility that it will not do so. [11]	Include confirmation message that an action has been successfully completed where there is a significant possibility that it will not do so. [11]
	*Timeout*	Timeout during answering must be permitted. [11]	Timeout during answering must be permitted. [6]	Timeout during answering must be permitted. [6]	Timeout during answering must be permitted. [6]
	*Progress indicators*	Use a progress indicator in order to reduce respondent loss. [6] Insert words and/or symbols that accurately communicate progress towards completion, in order to avoid premature termination. [5] Insert a simple progress indicator to show users how far they are through the sequence, especially for long quality of life questionnaires. [16] Regulate the progress process, either by making navigation decisions automatically on the basis of data already available within the application, or by presenting the options in a form that is completely straightforward for the patient. [11] Design progress process in a way that the user does not consider it as an endless processing. [11] Give users the information concerning the duration of the questionnaire: it should be clear to how many questions remain to be answered. [6] Place the navigational buttons at the bottom of the screen, ensuring that the user would have to scroll down the side in order to read all of the information on a page. [14]	Use a progress indicator in order to reduce respondent loss. [18] Insert words and/or symbols that accurately communicate progress towards completion, in order to avoid premature termination. [5] Insert a simple progress indicator to show users how far they are through the sequence, especially for long quality of life questionnaires. [16] Regulate the progress process, either by making navigation decisions automatically on the basis of data already available within the application, or by presenting the options in a form that is completely straightforward for the patient. [11] Design progress process in a way that the user does not consider it as an endless processing. [11] Give users the information concerning the duration of the questionnaire: it should be clear to how many questions remain to be answered. [6] Place the navigational buttons at the bottom of the screen, ensuring that the user would have to scroll down the side in order to read all of the information on a page. [14]	Use a progress indicator in order to reduce respondent loss. [18] Insert words and/or symbols that accurately communicate progress towards completion, in order to avoid premature termination. [5] Insert a simple progress indicator to show users how far they are through the sequence, especially for long quality of life questionnaires. [16] Regulate the progress process, either by making navigation decisions automatically on the basis of data already available within the application, or by presenting the options in a form that is completely straightforward for the patient. [11] Design progress process in a way that the user does not consider it as an endless processing. [11] Give users the information concerning the duration of the questionnaire: it should be clear to how many questions remain to be answered. [6] Place the navigational buttons at the bottom of the screen, ensuring that the user would have to scroll down the side in order to read all of the information on a page. [14]	Use a progress indicator in order to reduce respondent loss. [18] Insert words and/or symbols that accurately communicate progress towards completion, in order to avoid premature termination. [5] Insert a simple progress indicator to show users how far they are through the sequence, especially for long quality of life questionnaires. [16] Regulate the progress process, either by making navigation decisions automatically on the basis of data already available within the application, or by presenting the options in a form that is completely straightforward for the patient. [11] Design progress process in a way that the user does not consider it as an endless processing. [11] Give users the information concerning the duration of the questionnaire: it should be clear to how many questions remain to be answered. [6] Place the navigational buttons at the bottom of the screen, ensuring that the user would have to scroll down the side in order to read all of the information on a page. [14]
	*One page and multiple page design*	Think carefully to which type of page design choose: one-page design reflects the conventional formula where there is no interaction with the respondent during questionnaire completion; on the other hand multiple-page design increases the controls on non response questions, and also increases the risk of abandoning the questionnaire. [18] Try to include only one question for page; if bandwidth is not enough, design questionnaire flow so that the users’ attention is not diverted by other cognitive processes. [2]	Think carefully to which type of page design choose: one-page design reflects the conventional formula where there is no interaction with the respondent during questionnaire completion; on the other hand multiple-page design increases the controls on non response questions, and also increases the risk of abandoning the questionnaire. [14] Try to include only one question for page; if bandwidth is not enough, design questionnaire flow so that the users’ attention is not diverted by other cognitive processes. [2]	Think carefully to which type of page design choose: one-page design reflects the conventional formula where there is no interaction with the respondent during questionnaire completion; on the other hand multiple-page design increases the controls on non response questions, and also increases the risk of abandoning the questionnaire. [14] Try to include only one question for page; if bandwidth is not enough, design questionnaire flow so that the users’ attention is not diverted by other cognitive processes. [2]	Think carefully to which type of page design choose: one-page design reflects the conventional formula where there is no interaction with the respondent during questionnaire completion; on the other hand multiple-page design increases the controls on non response questions, and also increases the risk of abandoning the questionnaire. [14] Try to include only one question for page; if bandwidth is not enough, design questionnaire flow so that the users’ attention is not diverted by other cognitive processes. [2] Consider whether to use paging of questions to separate pages or one page questionnaires. In the case of online questionnaires, paging causes undesirable download delays. For touch screens we found it better to avoid paging due to these delays, since scrolling is easy. On the other hand, for conventional screens, the tradeoff between download delays and more cumbersome interaction needs to be considered. [14]
	*Phraseology*	Question Should focus on a single construct. [17] Avoid absolute terms, abbreviations and complex terminology. [9] The perspective from which questions should be addressed must be clarified, particularly for questions about attitudes. [9] Every question stem should include a clear request for either single or multiple responses and indicate the desired notation. [9] Questions must be formulated in a way that minimizes the need to reread portions in order to comprehend to response task. [9] Questions should contain no more than 20 words, and must be composed by familiar and simple words, and precise, consistent, grammatically correct sentences. [16] In questions’ composition avoid: acronyms, abbreviations, slang, jargon, proverbs, technical expressions, colloquial expressions, popular sayings, long alternatives as possible responses, different alternatives that could all be true, words which imply different meaning to different people, a lot of information carrying words, and loaded or provocative words. [12] Questions should be phrased so that they avoid being: double-barrelled; double-negative; hypothetical; negative; leading or biasing; two-edged. [12] Compose questions in the present tense using the active voice. [12] Explain users the meanings initials or acronyms. [12] Use general standards for the correct formulation of questions. [12] Write concise and precisely formulated questions that can be understood in the same way by all respondents. [2] Question’s wording should not try respondents’ abilities: do not ask the user to answer questions on concepts unknown to him. [14] One question must not contain too many concepts. [14] Questions should correspond to the thematic reference. [14] Do not use question structures that have known measurement problems on paper questionnaires. [14] Limit the amount of text in the question, in order to have more space for answer categories and avoid scrolling. [14] Don’t state your scale in the question. [10] Remove superfluous words: get to the point. [10]	Question Should focus on a single construct. [10] Avoid absolute terms, abbreviations and complex terminology. [9] The perspective from which questions should be addressed must be clarified, particularly for questions about attitudes. [9] Every question stem should include a clear request for either single or multiple responses and indicate the desired notation. [9] Questions must be formulated in a way that minimizes the need to reread portions in order to comprehend to response task. [9] Questions should contain no more than 20 words, and must be composed by familiar and simple words, and precise, consistent, grammatically correct sentences. [16] In questions’ composition avoid: acronyms, abbreviations, slang, jargon, proverbs, technical expressions, colloquial expressions, popular sayings, long alternatives as possible responses, different alternatives that could all be true, words which imply different meaning to different people, a lot of information carrying words, and loaded or provocative words. [12] Questions should be phrased so that they avoid being: double-barrelled; double-negative; hypothetical; negative; leading or biasing; two-edged. [12] Compose questions in the present tense using the active voice. [12] Explain users the meanings initials or acronyms. [12] Use general standards for the correct formulation of questions. [12] Write concise and precisely formulated questions that can be understood in the same way by all respondents. [2] Question’s wording should not try respondents’ abilities: do not ask the user to answer questions on concepts unknown to him. [14] One question must not contain too many concepts. [14] Questions should correspond to the thematic reference. [14] Do not use question structures that have known measurement problems on paper questionnaires. [14] Limit the amount of text in the question, in order to have more space for answer categories and avoid scrolling. [14] Don’t state your scale in the question. [10] Remove superfluous words: get to the point. [10]	Question Should focus on a single construct. [10] Avoid absolute terms, abbreviations and complex terminology. [9] The perspective from which questions should be addressed must be clarified, particularly for questions about attitudes. [9] Every question stem should include a clear request for either single or multiple responses and indicate the desired notation. [9] Questions must be formulated in a way that minimizes the need to reread portions in order to comprehend to response task. [9] Questions should contain no more than 20 words, and must be composed by familiar and simple words, and precise, consistent, grammatically correct sentences. [16] In questions’ composition avoid: acronyms, abbreviations, slang, jargon, proverbs, technical expressions, colloquial expressions, popular sayings, long alternatives as possible responses, different alternatives that could all be true, words which imply different meaning to different people, a lot of information carrying words, and loaded or provocative words. [12] Questions should be phrased so that they avoid being: double-barrelled; double-negative; hypothetical; negative; leading or biasing; two-edged. [12] Compose questions in the present tense using the active voice. [12] Explain users the meanings initials or acronyms. [12] Use general standards for the correct formulation of questions. [12] Write concise and precisely formulated questions that can be understood in the same way by all respondents. [2] Question’s wording should not try respondents’ abilities: do not ask the user to answer questions on concepts unknown to him. [14] One question must not contain too many concepts. [14] Questions should correspond to the thematic reference. [14] Do not use question structures that have known measurement problems on paper questionnaires. [14] Limit the amount of text in the question, in order to have more space for answer categories and avoid scrolling. [14] Don’t state your scale in the question. [10] Remove superfluous words: get to the point. [10]	Question Should focus on a single construct. [10] Avoid absolute terms, abbreviations and complex terminology. [9] The perspective from which questions should be addressed must be clarified, particularly for questions about attitudes. [9] Every question stem should include a clear request for either single or multiple responses and indicate the desired notation. [9] Questions must be formulated in a way that minimizes the need to reread portions in order to comprehend to response task. [9] Questions should contain no more than 20 words, and must be composed by familiar and simple words, and precise, consistent, grammatically correct sentences. [16] In questions’ composition avoid: acronyms, abbreviations, slang, jargon, proverbs, technical expressions, colloquial expressions, popular sayings, long alternatives as possible responses, different alternatives that could all be true, words which imply different meaning to different people, a lot of information carrying words, and loaded or provocative words. [12] Questions should be phrased so that they avoid being: double-barrelled; double-negative; hypothetical; negative; leading or biasing; two-edged. [12] Compose questions in the present tense using the active voice. [12] Explain users the meanings initials or acronyms. [12] Use general standards for the correct formulation of questions. [12] Write concise and precisely formulated questions that can be understood in the same way by all respondents. [2] Question’s wording should not try respondents’ abilities: do not ask the user to answer questions on concepts unknown to him. [14] One question must not contain too many concepts. [14] Questions should correspond to the thematic reference. [14] Do not use question structures that have known measurement problems on paper questionnaires. [14] Limit the amount of text in the question, in order to have more space for answer categories and avoid scrolling. [14] Don’t state your scale in the question. [10] Remove superfluous words: get to the point. [10]
	*Language and wording*	Pay attention to the language, because it influences directly the response formats, and so the response rate. [10] Present all information in the patient’s language, including any warning screens that may appear. Remember that some languages are more verbose than others. [9] Questions must be formulated in a socially and culturally sensitive manner, nonjudgmental and unbiased. [11] Use neutral tone for questions requesting users to rank items or elicit their opinions. [9] Use non-threatening language for provide missing item requests. [9] Additional information requested should be based on the patient’s own immediate situation. [16] Do not use inessential and inquisitive questions. [11] Asking for answer must be just in needful condition, although more questions lead to more perfect and clear information. [6] Use more indirect-oblique statements for questions on attitudes about particular topics. [6]	Pay attention to the language, because it influences directly the response formats, and so the response rate. [12] Present all information in the patient’s language, including any warning screens that may appear. Remember that some languages are more verbose than others. [9] Questions must be formulated in a socially and culturally sensitive manner, nonjudgmental and unbiased. [11] Use neutral tone for questions requesting users to rank items or elicit their opinions. [9] Use non-threatening language for provide missing item requests. [9] Additional information requested should be based on the patient’s own immediate situation. [16] Do not use inessential and inquisitive questions. [11] Asking for answer must be just in needful condition, although more questions lead to more perfect and clear information. [6] Use more indirect-oblique statements for questions on attitudes about particular topics. [6]	Pay attention to the language, because it influences directly the response formats, and so the response rate. [12] Present all information in the patient’s language, including any warning screens that may appear. Remember that some languages are more verbose than others. [9] Questions must be formulated in a socially and culturally sensitive manner, nonjudgmental and unbiased. [11] Use neutral tone for questions requesting users to rank items or elicit their opinions. [9] Use non-threatening language for provide missing item requests. [9] Additional information requested should be based on the patient’s own immediate situation. [16] Do not use inessential and inquisitive questions. [11] Asking for answer must be just in needful condition, although more questions lead to more perfect and clear information. [6] Use more indirect-oblique statements for questions on attitudes about particular topics. [6]	Pay attention to the language, because it influences directly the response formats, and so the response rate. [12] Present all information in the patient’s language, including any warning screens that may appear. Remember that some languages are more verbose than others. [9] Questions must be formulated in a socially and culturally sensitive manner, nonjudgmental and unbiased. [11] Use neutral tone for questions requesting users to rank items or elicit their opinions. [9] Use non-threatening language for provide missing item requests. [9] Additional information requested should be based on the patient’s own immediate situation. [16] Do not use inessential and inquisitive questions. [11] Asking for answer must be just in needful condition, although more questions lead to more perfect and clear information. [6] Use more indirect-oblique statements for questions on attitudes about particular topics. [6] Choose app questionnaire when the topic is personal and delicate. [12]
	*Multiple choice*	Double or triple banking of answer choices should be avoided. [15] Limitation of choices must be indicated (5 to 10 choices). [16] Use double-banking of answer when the number of answer choices exceeds the number that can be displayed in a single column on one screen. [6] Limit the number of answer categories. If is possible don’t exceed ten answer categories. [14] Limit the amount of text in each individual answer category. [10]	Double or triple banking of answer choices should be avoided. [10] Limitation of choices must be indicated (5 to 10 choices). [16] Use double-banking of answer when the number of answer choices exceeds the number that can be displayed in a single column on one screen. [6] Limit the number of answer categories. If is possible don’t exceed ten answer categories. [14] Limit the amount of text in each individual answer category. [10]	Double or triple banking of answer choices should be avoided. [10] Limitation of choices must be indicated (5 to 10 choices). [16] Use double-banking of answer when the number of answer choices exceeds the number that can be displayed in a single column on one screen. [6] Limit the number of answer categories. If is possible don’t exceed ten answer categories. [14] Limit the amount of text in each individual answer category. [10]	Double or triple banking of answer choices should be avoided. [10] Limitation of choices must be indicated (5 to 10 choices). [16] Use double-banking of answer when the number of answer choices exceeds the number that can be displayed in a single column on one screen. [6] Limit the number of answer categories. If is possible don’t exceed ten answer categories. [14] Limit the amount of text in each individual answer category. [10]
	*Skip/ Filter questions*	When skip patterns are used, apply a redundant visual change to highlight the questionnaire’s flow and gain user’s compliance. [10] Use skip questions to determine, on the basis of an individual respondent’s answer, which of the following questions the user should jump to when question path is response directed. [16] Make skip questions explicit, separate them from all other question types, include a training instruction, and use reminder instructions within the remainder of the questionnaire. [12] Use filter questions to increase the quality of data and avoid wrong answers. [12] Include skip directions to encourage users to mark the answers and click to the next applicable question. [14] Include a jump to page scroll bar at the top of every page, to make the users able to see how far along they are in the questionnaire flow, and to make them able to jump and change previous answers if necessary. [14]	When skip patterns are used, apply a redundant visual change to highlight the questionnaire’s flow and gain user’s compliance. [18] Use skip questions to determine, on the basis of an individual respondent’s answer, which of the following questions the user should jump to when question path is response directed. [16] Make skip questions explicit, separate them from all other question types, include a training instruction, and use reminder instructions within the remainder of the questionnaire. [12] Use filter questions to increase the quality of data and avoid wrong answers. [12] Include skip directions to encourage users to mark the answers and click to the next applicable question. [14] Include a jump to page scroll bar at the top of every page, to make the users able to see how far along they are in the questionnaire flow, and to make them able to jump and change previous answers if necessary. [14]	When skip patterns are used, apply a redundant visual change to highlight the questionnaire’s flow and gain user’s compliance. [18] Use skip questions to determine, on the basis of an individual respondent’s answer, which of the following questions the user should jump to when question path is response directed. [16] Make skip questions explicit, separate them from all other question types, include a training instruction, and use reminder instructions within the remainder of the questionnaire. [12] Use filter questions to increase the quality of data and avoid wrong answers. [12] Include skip directions to encourage users to mark the answers and click to the next applicable question. [14] Include a jump to page scroll bar at the top of every page, to make the users able to see how far along they are in the questionnaire flow, and to make them able to jump and change previous answers if necessary. [14]	When skip patterns are used, apply a redundant visual change to highlight the questionnaire’s flow and gain user’s compliance. [18] Use skip questions to determine, on the basis of an individual respondent’s answer, which of the following questions the user should jump to when question path is response directed. [16] Make skip questions explicit, separate them from all other question types, include a training instruction, and use reminder instructions within the remainder of the questionnaire. [12] Use filter questions to increase the quality of data and avoid wrong answers. [12] Include skip directions to encourage users to mark the answers and click to the next applicable question. [14] Include a jump to page scroll bar at the top of every page, to make the users able to see how far along they are in the questionnaire flow, and to make them able to jump and change previous answers if necessary. [14]
	*Invitation and reminders*		Use reminder letters and telephone contact, in order to increase response rates. [18] Use 3 follow up weaves: an initial reminder postcard sent 1 week after the initial mailing of the questionnaire to the entire sample, and 2 reminders (a letter plus replacement questionnaire) sent at 3 and 7 weeks to non respondent, with the final letter and replacement questionnaire sent by certified mail. [9] Send a pre-notification to prospective respondents in order to increase response rate and speed of response to survey. [9] Use the e-mail invitation in order to elicit responses to a Web survey. [5] Undertake multiple attempts to contact potential respondents, through pre notifications, reminders and replacement surveys, thank you notes. [5] Send an announcement prior to the survey, in order to increase response time. [5] The response rate can be increased through the use of random encouragement, namely a personal invitation via email to complete the survey. [5]	Use reminder letters and telephone contact, in order to increase response rates. [5] Use 3 follow up weaves: an initial reminder postcard sent 1 week after the initial mailing of the questionnaire to the entire sample, and 2 reminders (a letter plus replacement questionnaire) sent at 3 and 7 weeks to non respondent, with the final letter and replacement questionnaire sent by certified mail. [9] Send a pre-notification to prospective respondents in order to increase response rate and speed of response to survey. [9] Use the e-mail invitation in order to elicit responses to a Web survey. [5] Undertake multiple attempts to contact potential respondents, through pre notifications, reminders and replacement surveys, thank you notes. [5] Send an announcement prior to the survey, in order to increase response time. [5] The response rate can be increased through the use of random encouragement, namely a personal invitation via email to complete the survey. [5]	
	*Instructions*	Include operational definitions before potentially ambiguous questions. [5] Include clear instructions to skip non applicable questions. [9] Instructions must be placed exactly where the information is needed. [9] Include special instructions (only if those are essential) as a part of the question statement. [16] Separate optional or occasionally needed instructions from the question statement, using font or symbol instructions. [16] Include special guidance instruction in the normal flow of the questionnaire and not separate them from questions. [16] Help user to follow the questionnaire flow, placing instruction for determining eligibility for responding to a section or other major efforts inside navigational guides. [16] Unless a branching instruction depends on it, do not require users to provide an answer to each question before being allowed to answer any subsequent question. [16] Avoid excessive use of check all that apply questions because of the tendency of respondents to satisfied and choose earlier listed answer choices. [16] Definitions, explanations, and instructions in response path must be placed exactly where needed by the respondent. [16] Instruction must be constructed thinking about population need and characteristics. [16] Warnings of unreasonable values must be used only if necessary: avoid them when numeric options are presented as a multi-choice response. [16] If range or logic checks are necessary, include a message that state why there is a warning, and what the patient should do. [11] Make all necessary information simultaneously available on the screen, for both the questions and the response options. [11] Make clear the guidance context and indicate its place. [11] Explain technical instructions in an understandable way for non-technical people. [6] Make sure that all mechanisms for navigation are clearly identified and easy to find. [12] Make sure that answering instructions are extremely clear, in order to facilitate the completion of the questionnaire. [12] Include a sort of help desk in order to collect users’ difficulties with survey access, instructions, and other survey issues. [1] Include operational instructions as part of each question where the action is to be taken. [1] Include specific technical instructions at the point where they are needed. [20] Include understandable notes to help users answer the questions. [14]	Include operational definitions before potentially ambiguous questions. [14] Include clear instructions to skip non applicable questions. [9] Instructions must be placed exactly where the information is needed. [9] Include special instructions (only if those are essential) as a part of the question statement. [16] Separate optional or occasionally needed instructions from the question statement, using font or symbol instructions. [16] Include special guidance instruction in the normal flow of the questionnaire and not separate them from questions. [16] Help user to follow the questionnaire flow, placing instruction for determining eligibility for responding to a section or other major efforts inside navigational guides. [16] Unless a branching instruction depends on it, do not require users to provide an answer to each question before being allowed to answer any subsequent question. [16] Avoid excessive use of check all that apply questions because of the tendency of respondents to satisfied and choose earlier listed answer choices. [16] Definitions, explanations, and instructions in response path must be placed exactly where needed by the respondent. [16] Instruction must be constructed thinking about population need and characteristics. [16] Warnings of unreasonable values must be used only if necessary: avoid them when numeric options are presented as a multi-choice response. [16] If range or logic checks are necessary, include a message that state why there is a warning, and what the patient should do. [11] Make all necessary information simultaneously available on the screen, for both the questions and the response options. [11] Make clear the guidance context and indicate its place. [11] Include aids in the same place on each page of a questionnaire’s website. [6] Explain technical instructions in an understandable way for non-technical people. [12] Make sure that all mechanisms for navigation are clearly identified and easy to find. [12] Make sure that answering instructions are extremely clear, in order to facilitate the completion of the questionnaire. [12] The access to the survey must be easy: it is better to use a URL embedded with a unique identifier for each respondent. [1] Include a sort of help desk in order to collect users’ difficulties with survey access, instructions, and other survey issues. [1] Include operational instructions as part of each question where the action is to be taken. [1] Include specific technical instructions at the point where they are needed. [20] Include understandable notes to help users answer the questions. [14]	Include operational definitions before potentially ambiguous questions. [14] Include clear instructions to skip non applicable questions. [9] Instructions must be placed exactly where the information is needed. [9] Include special instructions (only if those are essential) as a part of the question statement. [16] Separate optional or occasionally needed instructions from the question statement, using font or symbol instructions. [16] Include special guidance instruction in the normal flow of the questionnaire and not separate them from questions. [16] Help user to follow the questionnaire flow, placing instruction for determining eligibility for responding to a section or other major efforts inside navigational guides. [16] Unless a branching instruction depends on it, do not require users to provide an answer to each question before being allowed to answer any subsequent question. [16] Avoid excessive use of check all that apply questions because of the tendency of respondents to satisfied and choose earlier listed answer choices. [16] Definitions, explanations, and instructions in response path must be placed exactly where needed by the respondent. [16] Instruction must be constructed thinking about population need and characteristics. [16] Warnings of unreasonable values must be used only if necessary: avoid them when numeric options are presented as a multi-choice response. [16] If range or logic checks are necessary, include a message that state why there is a warning, and what the patient should do. [11] Make all necessary information simultaneously available on the screen, for both the questions and the response options. [11] Make clear the guidance context and indicate its place. [11] Include aids in the same place on each page of a questionnaire’s website. [6] Explain technical instructions in an understandable way for non-technical people. [12] Make sure that all mechanisms for navigation are clearly identified and easy to find. [12] Make sure that answering instructions are extremely clear, in order to facilitate the completion of the questionnaire. [12] The access to the survey must be easy: it is better to use a URL embedded with a unique identifier for each respondent. [1] Include a sort of help desk in order to collect users’ difficulties with survey access, instructions, and other survey issues. [1] Include operational instructions as part of each question where the action is to be taken. [1] Include specific technical instructions at the point where they are needed. [20] Include understandable notes to help users answer the questions. [14]	Include operational definitions before potentially ambiguous questions. [14] Include clear instructions to skip non applicable questions. [9] Instructions must be placed exactly where the information is needed. [9] Include special instructions (only if those are essential) as a part of the question statement. [16] Separate optional or occasionally needed instructions from the question statement, using font or symbol instructions. [16] Include special guidance instruction in the normal flow of the questionnaire and not separate them from questions. [16] Help user to follow the questionnaire flow, placing instruction for determining eligibility for responding to a section or other major efforts inside navigational guides. [16] Unless a branching instruction depends on it, do not require users to provide an answer to each question before being allowed to answer any subsequent question. [16] Avoid excessive use of check all that apply questions because of the tendency of respondents to satisfied and choose earlier listed answer choices. [16] Definitions, explanations, and instructions in response path must be placed exactly where needed by the respondent. [16] Instruction must be constructed thinking about population need and characteristics. [16] Warnings of unreasonable values must be used only if necessary: avoid them when numeric options are presented as a multi-choice response. [16] If range or logic checks are necessary, include a message that state why there is a warning, and what the patient should do. [11] Make all necessary information simultaneously available on the screen, for both the questions and the response options. [11] Make clear the guidance context and indicate its place. [11] Include aids in the same place on each page of a questionnaire’s website. [6] Explain technical instructions in an understandable way for non-technical people. [12] Make sure that all mechanisms for navigation are clearly identified and easy to find. [12] Make sure that answering instructions are extremely clear, in order to facilitate the completion of the questionnaire. [12] The access to the survey must be easy: it is better to use a URL embedded with a unique identifier for each respondent. [1] Include a sort of help desk in order to collect users’ difficulties with survey access, instructions, and other survey issues. [1] Include operational instructions as part of each question where the action is to be taken. [1] Include specific technical instructions at the point where they are needed. [20] Include understandable notes to help users answer the questions. [14]
	*Anonymity and privacy*	Measures should be taken to ensure anonymity or at least confidentiality. [14] Survey identification procedures can be considered a breach of respondent anonymity, and thus negatively influencing response rate. It is difficult to achieve full anonymity. Ensure almost confidentially, by informing respondents that their email address will not be recorded with their survey responses, in addition to the fact that survey data will only be analyzed at the aggregate level. [5] Adopt clear, visible, respondent-friendly privacy policies. [5] Inform respondents about their privacy before starting questionnaires. [1]	Measures should be taken to ensure anonymity or at least confidentiality. [15] Survey identification procedures can be considered a breach of respondent anonymity, and thus negatively influencing response rate. It is difficult to achieve full anonymity. Ensure almost confidentially, by informing respondents that their email address will not be recorded with their survey responses, in addition to the fact that survey data will only be analyzed at the aggregate level. [5] Ensure that respondents’ privacy and perception of privacy are protected, especially by encrypting the survey data and preserving anonymity of data received. [5] Don’t contact respondents by email, unless they say it is all right. [12] Adopt clear, visible, respondent-friendly privacy policies. [1] Inform respondents about their privacy before starting questionnaires. [1]	Measures should be taken to ensure anonymity or at least confidentiality. [15] Survey identification procedures can be considered a breach of respondent anonymity, and thus negatively influencing response rate. It is difficult to achieve full anonymity. Ensure almost confidentially, by informing respondents that their email address will not be recorded with their survey responses, in addition to the fact that survey data will only be analyzed at the aggregate level. [5] Ensure that respondents’ privacy and perception of privacy are protected, especially by encrypting the survey data and preserving anonymity of data received. [5] Don’t contact respondents by email, unless they say it is all right. [12] Adopt clear, visible, respondent-friendly privacy policies. [1] Inform respondents about their privacy before starting questionnaires. [1]	Measures should be taken to ensure anonymity or at least confidentiality. [15] Survey identification procedures can be considered a breach of respondent anonymity, and thus negatively influencing response rate. It is difficult to achieve full anonymity. Ensure almost confidentially, by informing respondents that their contacts will not be recorded with their survey responses, in addition to the fact that survey data will only be analyzed at the aggregate level. [5] Ensure that respondents’ privacy and perception of privacy are protected, especially by encrypting the survey data and preserving anonymity of data received. [5] Adopt clear, visible, respondent-friendly privacy policies. [12] Inform respondents about their privacy before starting questionnaires. [1]
	*Costs*		Consider the fact that users must pay for the time they are online: it is better to distribute the questionnaire in a mixed mode design (email and paper-and-pencil). [15]	Consider the fact that users must pay for the time they are online: it is better to distribute the questionnaire in a mixed mode design (email and paper-and-pencil). [5]	Consider the fact that users must pay for the time they are online: it is better to distribute the questionnaire in a mixed mode design (email and paper-and-pencil). [5]
	*Repeated answers*		Check the email address of the respondents in order to omit repeated answers in data processing and analyzing. [5] If cookies are used to omit repeated answers, consider the possibility of a new source of non response, resulting from the exclusion of users who restrict placement of such cookies on their computers. [6] If IP number is used to omit repeated answer, consider that some computers may have a multi-user function, and Internet providers can assigne to users different unique identifiers for each dial-up session. [5] Include an automatic “Thank you” message after user has pressed the submit button, in order to avoid multiple press of such button and the multiple transmission of the questionnaire. [5] Check record time, date and addresses to avoid repeated answer sent from certain IP or in short time distance. [5]	If cookies are used to omit repeated answers, consider the possibility of a new source of non response, resulting from the exclusion of users who restrict placement of such cookies on their computers. [6] If IP number is used to omit repeated answer, consider that some computers may have a multi-user function, and Internet providers can assigne to users different unique identifiers for each dial-up session. [5] Include an automatic “Thank you” message after user has pressed the submit button, in order to avoid multiple press of such button and the multiple transmission of the questionnaire. [5] Check record time, date and addresses to avoid repeated answer sent from certain IP or in short time distance. [5]	If cookies are used to omit repeated answers, consider the possibility of a new source of non response, resulting from the exclusion of users who restrict placement of such cookies on their computers. [6] If IP number is used to omit repeated answer, consider that some computers may have a multi-user function, and Internet providers can assigne to users different unique identifiers for each dial-up session. [5] Include an automatic “Thank you” message after user has pressed the submit button, in order to avoid multiple press of such button and the multiple transmission of the questionnaire. [5] Check record time, date and addresses to avoid repeated answer sent from certain IP or in short time distance. [5]
	*Screen and type of device*	Use devices with 20 cm diagonal screens, if the survey is realized in a clinical setting. [6] When using an on-screen keypad, it is best to set this up a custom layout specifically for the purpose, using only relevant keys. The same principles applies for text keypads. In this way the keypad can be simplified, and the individual keys made larger. [11] Make sure to minimize the changes from keyboard entries to mouse entries, in order to not confuse the user. [11]	Make sure to minimize the changes from keyboard entries to mouse entries, in order to not confuse the user. [14]	Make sure to minimize the changes from keyboard entries to mouse entries, in order to not confuse the user. [14]	Make sure to minimize the changes from keyboard entries to mouse entries, in order to not confuse the user. [14] In mobile surveys remember to consider the four most important things: small screen size, data entry method and interaction style, mobile context and chosen implementation for the questionnaire. [14] Try to identify all devices supported in your data collection software: not only major brands but consider that there are many more and many models. [17] Minimize the skin to maximize the screen size/space. [10]
	*User’s computer competence*	Estimate the computer equipment respondents may be using as well as their competence with particular programs. [10] Provide a printable version of the questionnaire, if respondents need to prepare information before answering. [5] Build a system virtually usable by all patients eligible for the survey. [16] The data collection system should not exclude older patients, as a consequence of less familiarity with computer system, and a higher probability to have problems such as poor eyesight or tremor. [11] Build a system suitable for a wide range of patients, varying in physical and mental health status, age, and in their experience with technology. [11] Design the system with the less knowledgeable, low-end computer user in mind. [11] Provide an alternative printable format of the questionnaire, and of all referenced articles or documentation. [12] Questionnaires’ technical requirements should be tailored on the target group’s minimal technical requirements, particularly for file sizes, use of graphics and use of advanced programming. [12] Ensure that different devices with different user experiences do not affect the data. [14]	Ensure that potential respondents have access to electronic mail or the Internet. [10] Estimate the computer equipment respondents may be using as well as their competence with particular programs. [9] Web survey must be designed for those with older browser and poorer communication rather than those with state of the art access. [5] Provide a printable version of the questionnaire, if respondents need to prepare information before answering. [16] Build a system virtually usable by all patients eligible for the survey. [16] The data collection system should not exclude older patients, as a consequence of less familiarity with computer system, and a higher probability to have problems such as poor eyesight or tremor. [11] Build a system suitable for a wide range of patients, varying in physical and mental health status, age, and in their experience with technology. [11] Design the system with the less knowledgeable, low-end computer user in mind. [11] Provide an alternative printable format of the questionnaire, and of all referenced articles or documentation. [12] Questionnaires’ technical requirements should be tailored on the target group’s minimal technical requirements, particularly for file sizes, use of graphics and use of advanced programming. [12] Ensure that different devices with different user experiences do not affect the data. [14]	Ensure that potential respondents have access to electronic mail or the Internet. [10] Estimate the computer equipment respondents may be using as well as their competence with particular programs. [9] Web survey must be designed for those with older browser and poorer communication rather than those with state of the art access. [5] Provide a printable version of the questionnaire, if respondents need to prepare information before answering. [16] Build a system virtually usable by all patients eligible for the survey. [16] The data collection system should not exclude older patients, as a consequence of less familiarity with computer system, and a higher probability to have problems such as poor eyesight or tremor. [11] Build a system suitable for a wide range of patients, varying in physical and mental health status, age, and in their experience with technology. [11] Design the system with the less knowledgeable, low-end computer user in mind. [11] Provide an alternative printable format of the questionnaire, and of all referenced articles or documentation. [12] Questionnaires’ technical requirements should be tailored on the target group’s minimal technical requirements, particularly for file sizes, use of graphics and use of advanced programming. [12] Ensure that different devices with different user experiences do not affect the data. [14]	Ensure that potential respondents have access to electronic mail or the Internet. [10] Estimate the computer equipment respondents may be using as well as their competence with particular programs. [9] Web survey must be designed for those with older browser and poorer communication rather than those with state of the art access. [5] Provide a printable version of the questionnaire, if respondents need to prepare information before answering. [16] Build a system virtually usable by all patients eligible for the survey. [16] The data collection system should not exclude older patients, as a consequence of less familiarity with computer system, and a higher probability to have problems such as poor eyesight or tremor. [11] Build a system suitable for a wide range of patients, varying in physical and mental health status, age, and in their experience with technology. [11] Design the system with the less knowledgeable, low-end computer user in mind. [11] Provide an alternative printable format of the questionnaire, and of all referenced articles or documentation. [12] Questionnaires’ technical requirements should be tailored on the target group’s minimal technical requirements, particularly for file sizes, use of graphics and use of advanced programming. [12] Pay attention to the length of answer: users could have some difficult to write a lot of words with touch screen. [14] Pay attention to the type of Smartphone. [15] Ensure that different devices with different user experiences do not affect the data. [15]
	*Technical procedures*	Ensure that the rule of the thumb must not make burdensome the task. [10] Respondents must not have to reconfigure computers, switch browsers, or download software to complete the questionnaire. [5] Ensure that procedures are as convenient and unobtrusive as possible. [16] If possible, include a date- last- modified notification and a copyright notice in the introductory pages. [11] The total completion time of the questionnaire must be minimized. [12]	Ensure that the rule of the thumb must not make burdensome the task. [17] Prefer email software that offers transformation of URLs into direct links to the Web site, in order to reducing additional actions of the respondents. [5] Respondents must not have to reconfigure computers, switch browsers, or download software to complete the questionnaire. [5] To avoid that respondents have to click to another location, include the questionnaire, if it is short, into an e-mail message. [16] Ensure that procedures are as convenient and unobtrusive as possible. [16] If possible, include a date- last- modified notification and a copyright notice in the introductory pages. [11] Think about slow modem connection when you chose the download time. [12] The total completion time of the questionnaire must be minimized. [14]	Ensure that the rule of the thumb must not make burdensome the task. [17] Prefer email software that offers transformation of URLs into direct links to the Web site, in order to reducing additional actions of the respondents. [5] Respondents must not have to reconfigure computers, switch browsers, or download software to complete the questionnaire. [5] To avoid that respondents have to click to another location, include the questionnaire, if it is short, into an e-mail message. [16] Ensure that procedures are as convenient and unobtrusive as possible. [16] If possible, include a date- last- modified notification and a copyright notice in the introductory pages. [11] Think about slow modem connection when you chose the download time. [12] The total completion time of the questionnaire must be minimized. [14]	Ensure that the rule of the thumb must not make burdensome the task. [17] Respondents must not have to reconfigure devices, switch browsers, or download software to complete the questionnaire. [5] Ensure that procedures are as convenient and unobtrusive as possible. [16] If possible, include a date- last- modified notification and a copyright notice in the introductory pages. [11] Think about slow modem connection when you chose the download time. [12] The total completion time of the questionnaire must be minimized. [14]
	*Browser and file size*		To avoid that users may not be able to open the questionnaire file, send also a program file attached to the email message. [17] Maintain the file’s imagines as small as possible. [5] Not use browser-specific HTML extensions. [14] Avoid the use of Java and JavaScript, depending on the target population. [14] Design the questionnaire in order that respondents must be able to complete it without the use of a graphic browser if they use a textbased to obtain information from the Web. [14] Not require users to make alterations to their browser’s preferences. [14] Do not use Java Script for online survey. [14]	Not exceed the 60KB of text and graphic content of total web-site. [10] Maintain the file’s imagines as small as possible. [12] Not use browser-specific HTML extensions. [14] Avoid the use of Java and JavaScript, depending on the target population. [14] Design the questionnaire in order that respondents must be able to complete it without the use of a graphic browser if they use a textbased to obtain information from the Web. [14] Not require users to make alterations to their browser’s preferences. [14] Do not use Java Script for online survey. [14]	Maintain the file’s imagines as small as possible. [10] Not use browser-specific HTML extensions. [14] Design the questionnaire in order that respondents must be able to complete it without the use of a graphic browser if they use a textbased to obtain information from the Web. [14] Not require users to make alterations to their browser’s preferences. [14] Remember that different phone platforms have different implementation environments. [14] Do not use Java Script for online survey. [10]

**Table 3:** Reviewed papers distributed according to information and pertinence.

FIRST CATEGORY	SECOND CATEGORY	ELECTRONIC QUESTIONNAIRE	EMAIL QUESTIONNAIRE	WEB QUESTIONNAIRE	APP QUESTIONNAIRE
**SURVEY’S DEVELOPMENT**	*Item’s definition*	[10]	[9]	[9]	[9]
	*Sample selection*	[10]	[11]	[5] [5]	
	*Testing and pretesting*	[1] [9]	[11] [9] [6]	[14] [9] [12] [1] [13]	
	*Reliability test*	[14]	[9]	[9]	
	*Validity test*	[9]	[9]	[9]	
**QUESTIONNAIRE DESIGN**	*Profile the target audience*		[9]	[5] [5]	[14]
	*Quality of data*	[15]	[9]	[5] [16]	[15] [15]
	*Welcome page*	[10]	[9]	[9] [9] [16] [12] [1] [20]	
	*Open/ closed questions*		[14]	[5] [12] [2]	[14] [15]
	*Rank order questions*			[10]	
	*Categorical or nominal questions*			[12]	
	*Magnitude estimate questions*			[12]	
	*Ordinal questions*			[12]	
	*Likert scale questions*			[12]	
	*Indeterminate and other response options*	[12]	[9]	[9] [9]	
	*Open/ closed responses*	[12]	[9]	[9]	
**QUESTIONNAIRE LAYOUT**	*Questionnaire length*	[9]	[9] [9] [5]	[6] [9] [5] [16] [12] [14]	[18] [17]
	*Questionnaire structure and questions’ order*	[19]	[9] [9] [16]	[12] [9] [16] [12] [1] [20]	
	*Layout*	[14] [9]	[11] [9] [5]	[16] [9] [5] [16] [12] [14]	[18] [17]
	*Scrolling*	[10]		[11] [16] [12] [2]	
	*Font size and style*	[14] [9]	[11]	[9] [9] [12]	[13]
**QUESTIONNAIRE LAYOUT**	*Color*	[17]	[11]	[16] [16] [12]	
	*Questions’ graphic*		[14]	[16] [16] [13] [20]	
	*Answers’ graphic*	[14]	[11]	[16] [16] [12]	
	*Symbols and imagines*	[14]	[11] [16]	[6] [16] [6] [12] [2]	
	*Tables and frame*	[14]	[9]	[9] [9] [12]	
	*Visual analogue scale*	[14]			
	*Matrix questions*			[11] [12]	
	*Drop down boxes*	[14]		[11] [16]	
	*Radio buttons*	[12]		[11] [9] [5]	
	*Check buttons*	[12]		[11] [9]	
**INFORMATIC ACCESSIBILITY**	*Questionnaire distribution and login procedures*	[12] [5]	[6] [5]	[6] [5] [16] [6] [12]	[14]
	*Cover letter*	[10]	[9] [9]	[5] [9]	
	*Thank you page and user’s feedback*	[5]	[11] [5]	[12] [5]	[12]
	*Confirmation message*	[12]			
	*Timeout*		[11]	[6]	
	*Progress indicators*	[6]	[11]	[6] [5] [16] [6] [14]	
	*One page and multiple page design*			[18] [9]	[2] [2]
	* Phraseology*	[17]	[9] [9]	[16] [9] [16] [12] [2]	[14]
	* Language and wording*	[10] [9]	[11] [9]	[6] [9] [16] [6]	[12]
	* Multiple choice*		[15] [16]	[6] [16] [6]	[14]
**INFORMATIC ACCESSIBILITY**	*Skip/ Filter questions*		[10]	[16] [16] [12] [14]	
	*Invitation and reminders*		[18] [9]	[5]	
	*Instructions*	[5] [9]	[11] [9] [16]	[6] [9] [16] [6] [12] [1] [13] [20]	
	*Anonymity and privacy*		[14]	[5] [5] [12]	[1]
	*Costs*			[15]	
	*Repeated answers*		[5]	[6] [5]	
	*Screen and type of device*	[6]		[11]	[14] [17]
	*User’s computer competence*	[10]	[11] [9]	[5] [9] [5] [16] [12]	[14] [15]
	*Technical procedures*	[10]		[11] [5] [16] [12]	[14]
	*Browser and file size*		[17]	[5] [12]	[14]

**Table 4:** Evidence-based classification on the impact of the various indications as proposed in the paper.

**GUIDELINE (article)**	**TYPE OF QUESTIONNAIRE**	**EFFICACY (article)**
Use “other” response options to enhance response rates in self administered questionnaires, or use them during questionnaire testing, in order to identify new issues or to elaborate on closed response formats. [10]	Electronic Questionnaire E-mail Questionnaire Web Questionnaire	Responses like “Don’t know/not sure” and “would rather not say” aren’t influenced by the presence of a progress indicator: across all 69 non demographic items in the survey, the mean number of such responses is virtually identical in both versions (7.91 for the progress indicator version and 7.92 for the version with no progress indicator). [9]
Use a personalize cover letter to increases survey response rate. [21]	Electronic Questionnaire E-mail Questionnaire Web Questionnaire	The study shows how personalization of the email invitations increase the response rate of 8.6 and 7.8 percentage points in the two experiments conducted. [9] Using the salutation “Dear Forename” increased the odds of a response by almost 40% compared to using an impersonal salutation like “Dear Student”. [22] Impact of personalized salutation when inviting existing panel members to exit the panel : using a non personalized salutation increases the chances of a person leaving the panel by 1.4 times. [23] Highest response is obtained when personalized invitation came from a high power source (53.4) and the lowest when an impersonal invitation came from a neutral power source (40.1%). In the experiment a neutral power condition is composed by: From (name), (Strategy, Planning and Partnerships), The Open University; while a high power condition is composed by: From Professor (name), Pro-vice chancellor (Strategy, Planning and Partnerships), The Open University. [23] Effect of personalization invitation on response rate: • response rate = 53.51% among the sample assigned to the non personalization condition “Dear student”; • response rate = 92.91% among the sample assigned to the personalization condition “Dear first name, last name”. [23]
Provide an estimate of the time required to complete the questionnaire. [24]	Electronic Questionnaire E-mail Questionnaire Web Questionnaire	Survey length statement given in the email invitation is unable to significantly influence the response rates: • response rate = 59.10% among the sample assigned to the specific survey length condition; • response rate = 57.32% among the sample assigned to the vague survey length condition. [9]
Use reminder letters and telephone contact, in order to increase response rates. [24]	E-mail Questionnaire	The study describes three email surveys conducted among public health physician in the UK. Researchers sent the questionnaire to the potentially respondents, than sent reminders at three and seven weeks and contacted all non responders by telephone at eight weeks. Results show that the response rate of the third survey increases from 81% to 88% after the telephone reminder. [9] The paper describes the design of a Web survey to determine the use of the Internet in clinical practice by dental professionals. After the first email, three additional messages were sent to non respondents during the following three weeks. In the second and in the third follow up, the survey was directly included in the email message. In addition, while the initial messages and the first follow up messages were sent from a generic email account, the third and the second follow up were sent from the list owner’s account directly, with a personal request for a response to the survey. The response rate for surveys entered via the Web was 32.9% after the initial mailing, 50.2% after the first follow up, 57.1% after the second follow up and 64.4% after the third follow up. [25] The aim of the study is to determine the effect of a sequential mixed mode survey on response rate. Researchers distributed questionnaires among a sample of college bound high school students. In the first stage respondents were asked to respond via the Web, all respondents obtained a reminder by mail (without hard copy), also a random subset of them received phone reminders. In the second stage non-respondents were mailed a hard copy of the questionnaire in addition to the Web survey option, all respondents obtained two reminders by mail (a postcard, and a letter with a hard copy), also a random subset of them received phone incentives in form of 3$ McDonald’s gift certificates. Results show that the phone reminders positively affects response rate: the response rate among people who received no phone reminder was 17.8%, while the response rate among people who received a phone reminder was 30.3%. Also, results show that the use of incentives positively affects response rate: the response rate on or after day 35 with incentives is 31.5%, and without incentives 5.6%. [26]
The survey topic must be relevant to the target group. [27]	E-mail Questionnaire Web Questionnaire	Respondents who are more interested in the topic are more likely to provide a response to the open questions in comparison to respondents who are less interested (86.2% vs. 62% for the first question; 88.3% vs. 60.1% for the second question). [5] Respondents who are more interested in the topic are more likely to provide the follow up probes in comparison to respondents who are less interested (33.5% vs. 17.1% responded to the probe after the first questions; 16.1% vs. 4.7% responded to the probe after the second questions). [28] Respondents who are more interested in the topic are more likely to provide significantly more themes in comparison to respondents who are less interested (2.7% vs. 2.1% for the first question; 2.0% vs.1.5% for the second question). [28] Respondents who are more interested in the topic are more likely to elaborate significantly more often in comparison to respondents who are less interested (32.1% vs. 17.9% for the first question; 21.6% vs.13.9% for the second question). [28] Respondents who are more interested in the topic are more likely to provide significantly more words most of the time in comparison to respondents who are less interested (11.8% vs. 8.5% for the first question; 11.1% vs.8.2% for the second question). [28]
Use a semiautomatic login procedure, including an access code, in order to not decrease response rates and increase data quality. [28]	Electronic Questionnaire Web Questionnaire	Automatic login significantly increases response rates by nearly 5 percentage points over manual login, perhaps because it is less burdensome to sample members. [5] Study’s data show that using a manual login procedures: • does not decrease response rates (45.2% for automatic login condition vs. 51.6% for manual login condition); • increase the overall degree of data quality: almost 5% more completely filled out questionnaires in the manual condition compared to those in the automatic condition [29]
It is better to create a plain questionnaire than a fancy version of the same questionnaire, because a plain version increases response rate, the completeness and reduces completion time. [30]	E-mail Questionnaire Web Questionnaire	The study presents two experimental questionnaire: the “fancy” questionnaire, and the “plain” questionnaire, both with the same wording and question order. • 93% of respondents assigned to the plain questionnaires submitted their questionnaire, while 82% of those assigned to the fancy questionnaire submitted the questionnaire after completing it. • The plain questionnaire obtained a higher response rate (41.1%) compared to the fancy design (36.29%). [5]
Use a mixed-mode strategy, (electronic and pen-and-paper questionnaire), to reach respondents without access to the Internet. [31]	E-mail Questionnaire Web Questionnaire	The aim of the study is to determine the effect of response mode (online mode, simultaneous mixed mode or sequential mixed mode) on response rate among a sample of doctors undertaking clinical practice. Study’s design considers three different response mode: • Online mode: respondents received a personal invitation letter with login details and option to request paper copy; respondents were sent a reminder letter around three weeks later that again included login details. • Simultaneous mixed mode respondents received a personal invitation letter with paper copy, reply paid envelope and option to complete online the questionnaire; respondents were sent a reminder letter around three weeks later that contained login details and option to request paper copy. • Sequential mixed mode: respondents received a personal invitation letter login details and option to request paper copy; respondents were sent a reminder letter around three weeks later that contained paper copy, reply paid envelope and option to complete online. Results show that mixed mode strategies had a higher response rate: in fact, while the online mode had a response rate 12.95%, the simultaneous mixed mode had a response rate 19.7%, and the sequential mixed mode had a response rate 20.7%. Thus, the study demonstrates that the difference in response rate between the sequential and simultaneous modes is not statistically significant. [5]
Use a progress indicator in order to reduce respondent loss. [32] Insert words and/or symbols that accurately communicate progress towards completion, in order to avoid premature termination. [5]	Web Questionnaire	The presence of progress indicator could interfere on the mean time needed to complete the surveys. The mean time in minutes to complete the survey was significantly higher (*p=*.01) for the progress indicator version (22.7 minutes) than for the version with no progress indicator (19.8 minutes). However, the presence of a progress indicator does not influence completeness rate, despite time requested: 89.9 percent of who received a progress indicator completed the survey, compared with 86.4 percent of who did not receive a progress indicator. This difference does not reach statistical significance (*p=*.13). [16] Progress indicator could not significantly decrease the break-off rate: in the progress indicator group the break off rate is 11.27%, while it is 12.55% in the group that was shown nothing. [21] The aim of the study is to determine how respondent-friendly design principles (in terms of: structure of the first question, use of graphical symbols conveying point of completion, and use of double banking for multiple response questions) influence web questionnaire response rates and data quality. • Questionnaire 1: has a fully visible first question and double-banking answers choices, but does not have graphical symbols or words that convey a sense of where the respondent is in the completion progress. • Questionnaire 2: has a fully visible first question and graphical symbols or words that convey a sense of where the respondent is in the completion progress, but does not have double-banking answers choices. • Questionnaire 3: has double-banking answers choices and graphical symbols or words that convey a sense of where the respondent is in the completion progress, but does not have a fully visible first question. • Questionnaire 4: does not have a fully visible first question, neither double-banking answers choices, neither graphical symbols or words that convey a sense of where the respondent is in the completion progress. Study’s results shows that having a POC indicator has little or no effect on dropout rates: 13% of respondents without POC indicator did not complete the questionnaire, while 14% of respondents with POC indicator completed the questionnaire. [24]
Drop down boxes should be used only when answering process is simplified and should be identified with click here. [33] In compares to entry boxes, the use of radio buttons may decrease the likelihood of missing data. [16]	Web Questionnaire	In study’s second experiment, radio-buttons produce significantly lower drop-out rates than drop-down boxes: among students’ sample with fast internet connection the percentage of drop-out rate is 96.6% for respondents with radio-buttons, while drop-out rate is 86.4% for respondents with drop-down boxes. Study’s results indicate that radio-buttons act as an initial barrier filters to respondents with slower internet connections. But among those who are connected to the internet with sufficiently fast connections and are thus less influenced by downloading times, radio-buttons produce less dropout the drop-down boxes. As radio-buttons require more time to download, but are easier to use than drop-down boxes, the choice between the two response formats is not straightforward and must be guided by other concerns, such as sample composition. [9]
When create a voluntary survey, be careful to keep the questionnaire short. [34] Be careful to the length of the questionnaire, including a small number of short, well focused, understandable questions, avoiding to require excessive concentration. [16] Keep survey length short, in order to avoid that users speed through the survey to completion. [17]	Web Questionnaire App Questionnaire App Questionnaire	The aim of the study is to determine how questionnaire’s length and difficult influence dropout rate. The experimental design consisted in five different type of questionnaire distributed among the sample: • One questionnaire of 12 questions, which did not vary by difficult • Two easy questionnaires, one with 24 questions and one with 36 questions • Two difficult questionnaires, one with 24 questions and one with 36 questions Results show that the addition of 12 questions (6 minutes) to the easier version did not impact dropout rate significantly. However, the addition of 12 harder questions to an already difficult survey caused 6% of additional panelist to drop out. This suggests that the questionnaire’s difficult rather than questionnaire’s length impacts dropout rate. [19] The study examined respondents’ dropout rates among 1963 undergraduates participating in one of six survey based studies administered online. Studies ranged in length from 243 to 535 survey items, and measured constructs related to personality and emotion. • Study 1 with 243 items, obtained a response rate of 94.4% • Study 2 with 336 items, obtained a response rate of 85.4% • Study 3 with 365 items, obtained a response rate of 78.9% • Study 4 with 343 items, obtained a response rate of 75.4% • Study 5 with 535 items, obtained a response rate of 70.9% • Study 6 with 152 items, obtained a response rate of 71.6% Results’ analysis shows that among the total sample, 6% of participants dropped out after providing consent, and a total of 10.1% of participants discontinued within the first dozen responses. After the initial item set, dropout decelerated significantly, with a cumulative 13% of participants having dropped out after 100 responses, and 20.7% after 500 responses. [35] The aim of the study is to determine how the timing of follow-up, different incentives, length, and presentation of the questionnaire, influence the response rate and response quality in an online experimental setting. • Timing of the follow-up: a group received the reminder after one week, while the other group received the follow-up after two weeks; • Types of incentive: vouchers, donations or lottery; • Length of the questionnaire: long version (30-45 minutes) vs. short version (15-30 minutes); • Presentation of the questionnaire: textual vs. visual presentation. Response rates for different format and design parameters Timing of the follow up Type of Incentive Length of the questionnaire Presentation of the questionnaire *Early *21.2% *Late *19.5% *Voucher* 22.8% *Donation* 16.6% *Lottery *22.8% *Long *17.1% *Short *24.5% *Textual* 21.9% *Visual *19.0% The study shows there isn’t significant difference in response rate between the follow-up at 1 week (21.2%) and the follow up at 2 weeks (19.5). On the other hand, monetary interests (vouchers and lotteries) increase response rates (22.8%), in comparison to donations (16.6%). The shorter version of the questionnaire (24.5%) has a higher response rate than the long version (17.1%), while the response rate is significantly lower for the visual (19.0%) than for the textual version of the questionnaire (21.9), although this difference is relatively small. [36]
Design the questionnaire using a conventional formula similar to those normally used on paper self administered questionnaires. [37]	Web Questionnaire	In the Intervention Phase of the study, respondents were assigned to one of three questionnaire’s conditions, in order to determine how the use of prompts for speeding and non differentiation in grid questions have different impacts on respondent behavior. Experimental Conditions Behavior Triggering Intervention Intervention Message Displayed in a Pop-up Window No prompt (control) Non-differentation (ND) prompt: respondents prompted for non-differentiation, which includes straightlining (same responses for all the statements in a grid) and near straightlining (same responses for all the statements in a grid, except for one) When respondents straightline (same responses for all the items in a grid) or near-straightline (same responses except for one item) “You seem to have given very similar ratings for the different items in this question. Please think about each item on its own and be sure to give it enough thought so that your answer is informative and accurate. Do you want to go back and reconsider your answers?” (Yes/No) Speeding (SP) prompt: respondents prompted for speeding (response time<300 msec per word) When the total response time to the grid question is less than 300 msec per word (i.e., 0.3 sec multiplied by the number of words in the question) “You seem to have answered very quickly. Please be sure you have given all the items in the question sufficient thought so that your answer is informative and accurate. Do you want to go back and reconsider your answers?” (Yes/No) Results shows that both prompts reduce speeding and non differentiation behavior in grid questions: • Compared to control condition (98%), the speeding behavior is reduced by speeding prompt (96.8%) • Compared to control condition (35.5%), the speeding behavior is reduced by non differentiation prompt (30.2%) • Compared to control condition (47.1%), the near straightlining behavior is reduced by speeding prompt (49.8%) • Compared to control condition (62.6%), the near straightlining behavior is reduced by non differentiation prompt (55.3%) • Compared to control condition (48.1%), the straightlining behavior is reduced by speeding prompt (43.9%) • Compared to control condition (38%), the straightlining behavior is reduced by non differentiation prompt (23.4%) As we can see, although the grid design is widely used in Web questionnaires to present multiple items with the same response option, it does not’ improves answers’ quality. Compared to traditional Web surveys that are essentially online versions of paper questionnaires, an interactive Web survey interface may help respondents to give more truthful and correct answers. [16]
Double or triple banking of answer choices should be avoided. [38]	E-mail Questionnaire Web Questionnaire	The aim of the study is to determine how respondent-friendly design principles (in terms of: structure of the first question, use of graphical symbols conveying point of completion, and use of double banking for multiple response questions) influence web questionnaire response rates and data quality. • Questionnaire 1: has a fully visible first question and double-banking answers choices, but does not have graphical symbols or words that convey a sense of where the respondent is in the completion progress. • Questionnaire 2: has a fully visible first question and graphical symbols or words that convey a sense of where the respondent is in the completion progress, but does not have double-banking answers choices. • Questionnaire 3: has double-banking answers choices and graphical symbols or words that convey a sense of where the respondent is in the completion progress, but does not have a fully visible first question. • Questionnaire 4: does not have a fully visible first question, neither double-banking answers choices, neither graphical symbols or words that convey a sense of where the respondent is in the completion progress. Study’s results show that the double-banking treatments resulted in only 6% more checked items per respondent than for non-banked treatments. [16]
Insert a simple progress indicator to show users how far they are through the sequence, especially for long quality of life questionnaires. [33]	Electronic Questionnaire	The progress indicator show to significantly decrease the percentage of respondents saying that the survey took too long (6.25% vs. 13.48%). [11]
Use hyper text, colors, interaction strategies and other Web communication capabilities to create more attraction. [24]	E-mail Questionnaire Web Questionnaire	The study compare web survey ratings based on graphical and traditional standard web survey scales, including usability, engagement and enjoyment of taking part in the survey. Study’s results reports: • no significant disadvantages in completion time between graphical survey (10.8 minutes) and standard survey (9.7 minutes); • significant advantages in respondents’ perception of questionnaire’s usability in relation to the type of the survey (graphical v. standard): 86% of graphical sample find the question style to be enjoyable, compared to 72% of standard sample find the question style to be enjoyable; • significant advantages in respondent’s engagement in relation to the type of the survey (graphical vs. standard): the final question on the surveys asked respondents to include any open text comments they had on the survey itself, thus 33% respondents from the graphical survey added a comment, compared to only 20% from the standard survey. [6]
Adopt clear, visible, respondent-friendly privacy policies. [39]	Web Questionnaire	Paper’s study 2 reports an experimental manipulation of privacy and trust levels of the survey, presenting four different conditions: • Strong privacy condition: the first page of the web survey contained a strong privacy policy information • Weak privacy condition: the first page of the web survey contained a weak privacy policy information • High trust condition: institutional logo, no spelling mistakes and no advertisements • Low trust condition: advertisements for gambling and money transfer services and spelling and coding mistakes Privacy High Low Trust High 78.3% 82.1% Low 85.1% 60.4% Results’ study show that the impact of low privacy on self disclosure level is moderated by trust, such that high trust compensate for low privacy when examining response rates. The percentage of self disclosure is substantially reduced only when a weak privacy policy is combined with cues designed to reduce trust (60%). [1]
Think carefully to which type of page design choose: one page design reflects the conventional formula where there is no interaction with the respondent during questionnaire completion; on the other hand multiple-page design increases the controls on non response questions, and also increases the risk of abandoning the questionnaire. [40]	Web Questionnaire App Questionnaire	In the study, questionnaire’s completion time for the multiple-page design is 30% longer than for the one-page questionnaire. [2] The multiple-item-per-screen version took significantly less time (*p=*.01) to complete the 16 items than the single-item-per-screen version. [2]
The initial question must be fully visible on the first screen of the questionnaire, interest-getting, easily comprehended and answered by all respondents. Do not use drop-down box or require scrolling in order to see the entire first question. [21] At the first question include an item that is likely to be interesting, easy to answer, and fully visible on the first screen of the questionnaire. [20]	Web Questionnaire	The aim of the study is to determine how respondent-friendly design principles (in terms of: structure of the first question, use of graphical symbols conveying point of completion, and use of double banking for multiple response questions) influence web questionnaire response rates and data quality. • Questionnaire 1: has a fully visible first question and double-banking answers choices, but does not have graphical symbols or words that convey a sense of where the respondent is in the completion progress. • Questionnaire 2: has a fully visible first question and graphical symbols or words that convey a sense of where the respondent is in the completion progress, but does not have double-banking answers choices. • Questionnaire 3: has double-banking answers choices and graphical symbols or words that convey a sense of where the respondent is in the completion progress, but does not have a fully visible first question. • Questionnaire 4: does not have a fully visible first question, neither double-banking answers choices, neither graphical symbols or words that convey a sense of where the respondent is in the completion progress. Study’s results show that having the full first question visible has little or no effect on whether or not respondents decide to complete the question or continue with the survey. [14]
Choose a layout that don’t influence users’ opinions and answers. [33]	Web Questionnaire	The study tests the effects of repeated exposure to a certain image in a web survey on students’ housing conditions, showing four different type of image: • Image of upscale housing condition • Image of average housing condition • Image of deprived housing condition • No imagine The image was always located on the header of the questionnaire. The respective header appeared on top of each questionnaire page from the beginning and stayed the same throughout the whole survey. Questionnaire asked respondents to indicate personal satisfaction with current housing condition and rating of the city. • Interaction between type of image exposed and student’s satisfaction with current house situation: there is no significant evidence that the condition upscale and deprived produce contrast effects on satisfaction with the current housing condition (means of satisfaction = 4.31 for deprived image, and 4.31 for upscale image). • Interaction between type of image exposed and student’s rating of the city: there is no significant evidence that the condition upscale and deprived produce contrast effects on student’s city ratings (means of city ratings = 2.16 for deprived image, and 2.16 for upscale image). The study provides evidence on the phenomenon “banner blindness”, showing that header image is not overlooked completely by respondents, despite satisfaction and city ratings levels. In fact, in the open space for comments at the end of the survey, several respondents in the “deprived” and “upscale” conditions complained that the pictures were ridiculously bleak or too glamorous, respectively, and by no means representative of students’ housing conditions. [14] The images did indeed affect the self-ratings of health, producing lower ratings on average for the respondents who got the picture of the healthy woman (mean = 3.10) than those who got the picture of the sick woman (mean = 3.25). [41] When the imagine is inserted in the introduction or in the question area, we can find the hypothesized contrast effect, with respondents rating their health lower when exposed to the picture of the healthy woman (respectively 2.93 and 3.05), and higher when exposed to the picture of the sick woman (respectively 3.29 and 3.30). [42] When the imagine is inserted in the header of the questionnaire the difference between the two versions is not significant (mean 3.14 for sick woman, mean 3.29 for fit woman). One explanation for the lack of effect of the picture content in the header condition may be “banner blindness: when the image appears in the header area, it may either be ignored altogether (not seen) or viewed as irrelevant to the task. [42]
Try to include only one question for page; if bandwidth is not enough, design questionnaire flow so that the users’ attention is not diverted by other cognitive processes. [42]		The aim of the study is to determine the effect of questionnaire’s presentation (one web page vs. multiple web pages) on respondents’ preferences. Participants were asked to complete four questionnaires (BDI, BAI, MADRS and QOLI), presented wither with one entire questionnaire on one web page, or on multiple web pages. Hence, the questionnaire were either answered on a total of four web pages or on 83 web pages. Results show that a majority of participants in each of the ten diagnostic groups preferred the single item per page presentation format: • among the participant in the depressed group, over 90% of respondents for each group (MM, SS, MS, SM) preferred one item to a page; • among the participant in the panic disorder group, over 70% of respondents for each group (SM,MS) preferred one item to a page; • among the participant in the social phobia group, over 60% of respondents for each group (SM,MS,MM,SS) preferred one item to a page. [14]
In mobile surveys remember to consider the four most important things: small screen size, data entry method and interaction style, mobile context and chosen implementation for the questionnaire. [43]	App Questionnaire	Small screen can affect responding. In the survey, when response options extended beyond the screen, some respondents (23%) reported not having seen them. When part of the question providing strikingly different information extended beyond the screen, some respondents (5%) seemed to use only the first part of the question. [17]
Choose app questionnaire when the topic is personal and delicate. [44]	App Questionnaire	The aim of the study is to determine how the type of device influences completion rate and quality of response in questionnaires with sensitive topics. In the experiment, a questionnaire with sensitive topics was distributed in two consecutives waves. In the first wave, one part of the sample must complete the questionnaire using PC browser, while the rest of the sample must complete it on a mobile browser. In the second waves, those who participated in the mobile survey in the first wave must complete the questionnaire via PC, and vice versa. Results show that the type of device influences completion rate: • In the first wave, the completion rate to the PC survey (73.8%) is significantly higher than in the mobile survey (31%). Also, the break off rate in the mobile questionnaire (13.6%) is five times higher compared to the PC questionnaire (2.9%). • At the same way, in the second wave, the completion rate to the PC survey (88.4%) is significantly higher than in the mobile survey (38%). Also, the break off rate in the mobile questionnaire (12.7%) is 12 percentage points higher compared to the PC questionnaire (1%). Also study’s results show that 45% of mobile respondents complete the survey outside the home, compared to 29% of PC respondents. Those completing the survey on a mobile device reported lower levels of perceived privacy (63%) than those who completed it on a PC (75%). Thus, study shows that users tend to trust in data confidentially more when they complete the questionnaire on a PC rather than via cell phone. [15]

## LIST OF ABBREVIATIONS

WebSM: = (Web Survey Methodology)
PRO: = (Patient Reported Oucomes (P.R.O)

## References

[1] Evans J.R., Mathur A. (2005). The value of online surveys.. Intest. Res..

[2] Manfreda KL, Batagelj Z, Vehovar V (2002). Design of web survey questionnaires: Three basic experiments.. J. Comp. Med. Commun..

[3] Palmblad M., Tiplady B. (2004). Electronic diaries and questionnaires: Designing user interfaces that are easy for all patients to use.. Qual. Life Res..

[4] Lumsden J. (2005). Guidelines for the design of online-questionnaires.

[5] Van S.M., Jankowski N. (2006). Conducting Online Surveys.. Qual. Quant..

[6] Kalantari D.H., Kalantari D.E., Maleki S. (2011). E-survey (surveys based on e-mail & web).. Procedia Comput. Sci..

[7] Dillman D.A., Tortora R.D., Bowker D. (1998). Principles for constructing web surveys..

[8] Litwin M.S., Fink A. (1995). How to measure survey reliability and validity.. Sage.

[9] Burns K.E., Duffett M., Kho M.E., Meade M.O., Adhikari N.K., Sinuff T., Cook D.J., ACCADEMY Group (2008). A guide for the design and conduct of self-administered surveys of clinicians.. CMAJ.

[10] Bailey J., Bensky E., Link M. (2011). Can Your Smartphone Do This?: A New Methodology for Advancing Digital Ethnography. 2011.. In American Association for Public Opinion Research annual conference.

[11] Palmblad M., Tiplady B. (2004). Electronic diaries and questionnaires: Designing user interfaces that are easy for all patients to use.. Qual. Life Res..

[12] Lumsden J. (2005). Guidelines for the Design of Online-Questionnaires.. NRC Publications Archive.

[13] Lumsden J., Morgan W. Online-questionnaire design: Establishing guidelines and evaluating existing eupport.. http://nparc.cisti-icist.nrc-cnrc.gc.ca/ npsi/ctrl?action=rtdoc&an=8913785&lang=en.

[14] Batinic B., Reips U.D., Bosnjak M. (2002). Online social sciences..

[15] Dube S.R., Hu S.S., Fredner-Maguire N., Dayton J., Association A S (2012). A Focus Group Pilot Study of Use of Smartphone to Collect Information about Health Behaviors.. 67th Annual Conference of the American Association for Public Opinion Research (AAPOR).

[16] Dillman D.A., Smyth J.D., Christian L.M. (2008). Internet, Mail, and Mixed-Mode Surveys: The Tailored Design Method..

[17] Väätäjä H., Roto V. (2010). Mobile questionnaires for user experience evaluation.. CHI’10..

[18] Abraham S.Y., Steiger D.M., Sullivan C. (1998). Electronic and mail self-administered questionnaires: A comparative assessment of use among elite Populations.. Proceedings of the Section on Survey Research Methods.

[19] Emde M., Fuchs M. (2012). Using adaptive questionnaire design in open-ended questions: A field experiment.. 67th Annual Conference.

[20] Dillman D.A., Tortora R.D., Bowker D. (1998). Principles for constructing web surveys..

[21] Couper M.P., Traugott M.W., Lamias M.J. (2001). Web survey design and administration.. Public Opin. Q..

[22] Heerwegh D., Vanhove T., Loosveldt G., Matthijs K. (2004). Effects of personalization on web survey response rates and data quality..

[23] Joinson A.N., Reips U.D. (2007). Personalized salutation, power of sender and response rates to Web-based surveys.. Comput. Human Behav..

[24] Heerwegh D, Loosveldt G. (2006). An experimental study on the effects of personalization, Survey Length Statements, Progress Indicators, and survey Sponsor Logos in Web Surveys. J. Off Stat..

[25] Fischbacher C., Chappel D., Edwards R., Summerton N. (2000). Health surveys *via* the Internet: Quick and dirty or rapid and robust?. J. R. Soc. Med..

[26] Schleyer T.K., Forrest J.L. (2000). Methods for the design and administration of web-based surveys.. J. Am. Med. Inform. Assoc..

[27] Schonlau M., Asch B.J., Du C. (2003). Web surveys as part of a mixed-mode strategy for populations that cannot be contacted by E-Mail.. Soc. Sci. Comput. Rev..

[28] Holland J.L., Christian L.M. (2009). The Influence of topic interest and interactive probing on responses to open-ended questions in web surveys.. Soc. Sci. Comput. Rev..

[29] Crawford S.D., Couper M.P., Lamias M.J. (2001). Web surveys: perceptions of burden.. Soc. Sci. Comput. Rev..

[30] Heerwegh D., Loosveldt G. (2002). Web Surveys: The Effect of Controlling Survey Access Using PIN Numbers.. Soc. Sci. Comput. Rev..

[31] Dillman D.A., Tortora R.D., Conradt J., Bowker D., Association A.S. (1998). Influence of Plain *Vs.* Fancy Design on Response Rates for Web Surveys.. Joint Statistical Meetings..

[32] Scott A., Jeon S-H., Joyce C.M., Humphreys J.S., Kalb G., Witt J., Leahy A. (2011). A randomised trial and economic evaluation of the effect of response mode on response rate, response bias, and item non-response in a survey of doctors.. BMC Med. Res. Methodol..

[33] Healey B., Macpherson T., Kuijten B. (2005). An Empirical Evaluation of Three Web Survey Design Principles Market Bullet.

[34] Heerwegh D., Loosveldt G. (2002). An Evaluation of the effect of response formats on data quality in web surveys.. Soc. Sci. Comput. Rev..

[35] Burdein I. (2014). Shorter Isn’t Always Better. In: 2013 CASRO Online Research Conference..

[36] Hoerger M. (2010). Participant dropout as a function of survey length in internet-mediated university studies: Implications for study design and voluntary participation in psychological research.. Cyberpsychol. Behav. Soc. Netw..

[37] Deutskens E., de Ruyter K., Wetzels M., Oosterveld P. (2004). Response rate and response quality of internet-based surveys: Experiment Study.. Mark. Lett..

[38] Zhang C. (2013). Satisficing in Web Surveys: Implications for Data Quality and Strategies for Reduction..

[39] Stanley N., Jenkins S. (2007). Watch what I do: Using graphical input controls in Web surveys.. Challanges of a changing world..

[40] Joinson A.N., Reips U-D., Buchanan T., Schofield C.B. (2010). Privacy, Trust, and Self-Disclosure.. Hum. Comput. Interact..

[41] Barth A. (2014). Does the Choice of Header Images influence Responses? Findings from a Web Survey on Students’ Housing Situation.. Survey Methods: Insights from the Field..

[42] Couper M.P., Conrad F.G., Tourangeau R. (2007). Visual context effects in web surveys.. Public Opin. Q..

[43] Thorndike F.P., Carlbring P., Smyth F.L. (2009). Web-based measurement: Effect of completing single or multiple items per webpage.. Comput. Human Behav..

[44] Peytchev A., Hill C.A. (2010). Experiments in Mobile Web Survey Design: Similarities to other modes and unique considerations.. Soc. Sci. Comput. Rev..

[45] Heerwegh D., Loosveldt G. (2002). Web surveys: The effect of controlling survey access using PIN numbers.. Soc. Sci. Comput. Rev..

[46] Timmins F. (2015). Surveys and questionnaires in nursing research.. Nurs. Stand..

[47] Thom B. (2007). Role of the simple, self-designed questionnaire in nursing research.. J. Pediatr. Oncol. Nurs..

